# The Robo4 cytoplasmic domain is dispensable for vascular permeability and neovascularization

**DOI:** 10.1038/ncomms13517

**Published:** 2016-11-24

**Authors:** Feng Zhang, Claudia Prahst, Thomas Mathivet, Laurence Pibouin-Fragner, Jiasheng Zhang, Gael Genet, Raymond Tong, Alexandre Dubrac, Anne Eichmann

**Affiliations:** 1 Cardiovascular Research Center, Yale University School of Medicine, Department of Internal Medecine Cardiology, New Haven, Connecticut 06510-3221, USA; 2 INSERM U970, Paris Center for Cardiovascular Research (PARCC), 75015 Paris, France; 3 Genentech Inc., Molecular Oncology Division, South San Francisco, California 94030, USA; 4 Department of Cellular and Molecular Physiology, Yale University School of Medicine, New Haven, Connecticut 06510-3221, USA

## Abstract

Vascular permeability and neovascularization are implicated in many diseases including retinopathies and diabetic wound healing. Robo4 is an endothelial-specific transmembrane receptor that stabilizes the vasculature, as shown in *Robo4*^*−/−*^ mice that develop hyperpermeability, but how Robo4 signals remained unclear. Here we show that *Robo4* deletion enhances permeability and revascularization in oxygen-induced retinopathy (OIR) and accelerates cutaneous wound healing. To determine Robo4 signalling pathways, we generated transgenic mice expressing a truncated Robo4 lacking the cytoplasmic domain (Robo4ΔCD). Robo4ΔCD expression is sufficient to prevent permeability, and inhibits OIR revascularization and wound healing in *Robo4*^*−/−*^ mice. Mechanistically, Robo4 does not affect Slit2 signalling, but Robo4 and Robo4ΔCD counteract Vegfr2-Y949 (Y951 in human VEGFR2) phosphorylation by signalling through the endothelial UNC5B receptor. We conclude that Robo4 inhibits angiogenesis and vessel permeability independently of its cytoplasmic domain, while activating VEGFR2-Y951 via ROBO4 inhibition might accelerate tissue revascularization in retinopathy of prematurity and in diabetic patients.

## Abstract (web summary)

Robo4 is a transmembrane protein that regulates vascular permeability. Zhang *et al*. now reveal the mechanism of Robo4 action and show that Robo4 and UncB are required for VEGF-mediated regulation of vascular barrier by suppressing VEGF-induced phosphorylation of its receptor Vegfr2 on Y949.

## Graphical abstract

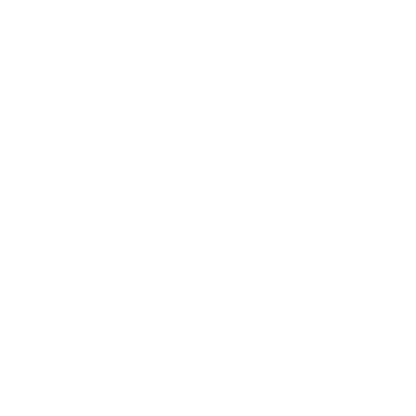

ROBO4 was identified 15 years ago in a bioinformatics search for endothelial-specific transcripts and initially named Magic Roundabout, to denote its homology to Robo receptors and its selective expression on endothelial cells in actively growing tumour vessels^1^. Robo4 encodes a 140 kDa protein that comprises a 60 kDa extracellular domain (ECD) with two immunoglobulin (Ig)-like domains and two fibronectin-repeat regions, a transmembrane domain and an intracellular domain (ICD) devoid of known catalytic sequences^2^. Robo4 is upregulated in endothelium of embryonic blood vessels and in micro-vessels of patients with tumours and vascular injury, but is also seen in quiescent endothelium^1^,^2^,^3^,^4^,^5^,^6^,^7^. Its endothelial-specific expression is regulated by activator protein-1 (AP-1) binding to a 3 kb fragment of the promoter^7^,^8^,^9^. Besides endothelial cells, Robo4 is also expressed in hematopoietic stem cells and regulates their trafficking^10^,^11^,^12^,^13^. The highly endothelial-specific Robo4 expression has prompted considerable interest in this molecule to selectively target or image tumour vascularization^9^,^14^,^15^,^16^.

*Robo4*^*−/−*^ mice are viable and fertile, demonstrating that Robo4 function is dispensable for developmental angiogenesis^17^,^18^. Adult *Robo4*^*−/−*^ mice exhibit enhanced corneal neovascularization induced by VEGF implantation in the eyes, and *Robo4* knockout breast tissue implanted into pregnant mice develops more vessels when compared with wild-type controls^18^,^19^, suggesting that Robo4 has anti-angiogenic effects in certain tissue contexts. In addition, Robo4 also maintains vascular barrier function, as shown in *Robo4*^*−/−*^ mice, which have increased VEGF-induced dermal permeability^17^,^18^. Furthermore *Robo4*^−/−^ mice show increased angiogenesis in oxygen-induced retinopathy (OIR)^17^. OIR is widely used to model ocular neovascular disease^20^,^21^. Neonatal mice are subjected to 75% oxygen exposure for 5 days (P7-P12), resulting in vascular obliteration. After being placed back to room air, OIR retinas enter into a proliferative phase, leading to re-growth of normal vessels (revascularization) and formation of pathological pre-retinal neovascular tufts driven by angiogenic factors. Thus, OIR models can be used to examine vessel loss, physiological revascularization and pathological neovascular tuft formation^20^,^21^.

VEGF signals permeability induction by activating a specific tyrosine residue in the VEGFR2 intracellular domain, Y951 (Y949 in mouse Vegfr2). The pY951 residue mediates binding of the T cell–specific adapter (TSAd), which is essential for VEGF-induced c-Src activation, vascular permeability and pathological angiogenesis^22^,^23^,^24^,^25^. Mutant mice carrying a substitution of Y949 to phenylalanine, and TSAd knockout mice are viable and fertile, but fail to show VEGF-induced Src phosphorylation and are deficient in VEGF- but not histamine-induced permeability *in vivo*^23^,^24^,^25^. They also show normal developmental angiogenesis, but decreased tumour vascularization^23^,^24^,^25^.

How Robo4 signals to maintain vascular barrier function is currently unclear. Initial studies reported that Robo4 acts as a receptor for Slit family proteins (Slit1–3) that regulate crossover of axons at the mid-line of the developing central nervous system^2^,^17^,^26^,^27^,^28^,^29^ and also mediate cardiovascular development and angiogenesis^15^,^30^,^31^,^32^,^33^. Slit2 binding to Robo4 was proposed to counteract VEGF-driven angiogenesis and vascular permeability by signalling through the Robo4 ICD^17^ that can bind to paxillin and other cytoskeletal modifying proteins^2^,^34^,^35^. However, Slit2 does not bind to Robo4 in Biacore binding assays, and Slit2 binding to vasculature is unaltered in *Robo4*^*−/−*^ mice^14^,^18^. In fact, crystal structure analysis showed that the critical Slit-binding residues in the Robo1 and 2 extracellular domains are not conserved in mammalian Robo3 and 4 (refs 36, 37, 38, 39). Alternatively, Robo4 could affect angiogenesis by modulating Slit2 signalling through Robo1 and 2. A recent study showed that tamoxifen-inducible deletion of *Slit2* in postnatal mice leads to severe retinal angiogenesis defects, demonstrating potent pro-angiogenic functions for Slit2 (ref. 32). Tamoxifen-inducible deletion of *Robo2* on a *Robo1-*null background phenocopies the *Slit2* mutant defects, demonstrating that Slit2 provides pro-angiogenic signals via Robo1 and 2 (ref. 32). These *in vivo* studies support pro-angiogenic Slit effects previously observed *in vitro*^40^,^41^,^42^,^43^,^44^,^45^. ROBO1 and 4 can be co-immunoprecipitated from HUVEC lysates, suggesting that Robo4 could restrain angiogenesis by acting as a dominant negative regulator of Slit2 signalling through Robo1 and 2 (ref. 35).

A third model of Robo4 action is that it signals through UNC5B, another endothelial-specific guidance receptor^46^,^47^,^48^,^49^. High-throughput screening of >1,500 secreted proteins and ECD domains with a Robo4-ECD-domain fused to the Fc portion of human Immunoglobulin (Robo4-ECD-Fc) identified the UNC5B-ECD-Fc as the only Robo4-binding protein^18^. The Robo4 ECD binds to the UNC5B ECD with a Kd of 12 nM (ref. 18). Robo4-ECD-Fc binds to cells transfected with UNC5B, and Robo4 binding to blood vessels is lost in *Unc5B*^*−/−*^ mice, together providing strong evidence for a direct interaction between both receptors. The Robo4 ECD activates signalling through Unc5B, which inhibits Src activation downstream of Vegfr2, thereby attenuating VEGF-mediated sprouting angiogenesis^18^. These data suggest that the Robo4 ECD might be sufficient to mediate Robo4 actions in the vasculature.

To distinguish between the different possible modes of Robo4 action, we generated mice lacking the Robo4 cytoplasmic signalling domain. If this domain was endowed with signalling capacity, mice should develop a phenotype resembling *Robo4* knockouts. However, we find that the Robo4 cytoplasmic domain is dispensable for its effect on angiogenesis. We show here that *Robo4*^*−/−*^ mice have increased ocular permeability and revascularization when subjected to OIR and exhibit accelerated healing of cutaneous wounds. Robo4ΔCD expression inhibits OIR revascularization, vessel permeability and wound healing in Robo4 knockout mice. Signalling studies show that the ROBO4 ECD signals via the UNC5B ICD to prevent activation of VEGFR2 Y951. The data suggest that promoting Y951 activation via ROBO4 blockade might represent an opportunity to enhance ocular revascularization in retinopathy of prematurity and wound healing in diabetic patients.

## Results

### Generation of mice lacking the Robo4 cytoplasmic domain

**Figure 1:**
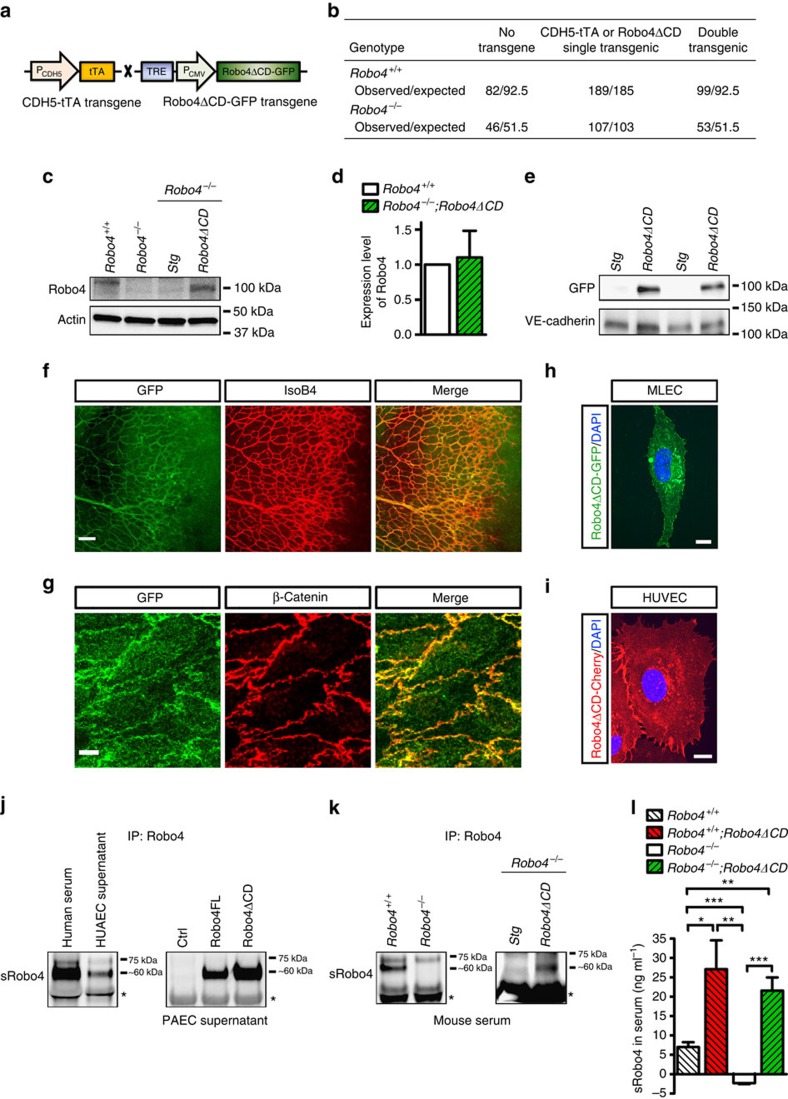
Generation and characterization of Robo4ΔCD transgenic mice. (**a**) Schematic of transgenic constructs. (**b**) Mendelian distribution of transgenic mice. 370 mice (53 litters) on *Robo4*^*+/+*^ background and 206 mice (31 litters) on *Robo4*^*−/−*^ background were genotyped. (**c**) Anti-Robo4 western blot on MLECs isolated from the indicated mice. Note lower molecular weight (∼100 kDa) of Robo4ΔCD-GFP than endogenous Robo4 (∼140 kDa). (**d**) Quantification of blots in **c**. *N*=3 mice per group. (**e**) Western blot with anti-GFP on mouse lung lysates. Each lane shows one lung lysate of the indicated genotype (all on *Robo4*^*+/+*^ background). (**f**) Vascular-specific anti-GFP staining of a P7 *Robo4*^*+/+*^*;Robo4ΔCD* retina counterstained with IsoB4. Scale bar, 100 μm. (**g**) En face anti-GFP staining of aortic endothelium of an adult *Robo4*^*+/+*^*;Robo4ΔCD* mouse counterstained with β-catenin. Scale bar, 20 μm. (**h**) Membrane GFP staining in *Robo4*^*+/+*^*;Robo4ΔCD* MLEC. (**i**) Membrane and junctional labelling of HUVECs infected with Robo4ΔCD-mCherry adenovirus. Scale bar, 10 μm. (**j**) Anti-Robo4 IP and western blot with human serum and cell culture supernatants from HUAECs and PAEC transfected with the indicated constructs. Note presence of a soluble Robo4 (sRobo4) band at ∼60 kDa. Lower bands (*) correspond to serum Ig. (**k**) SRobo4 is absent in *Robo4*^*−/−*^ and *Robo4*^*−/−*^*;Stg* mouse serum but detected in *Robo4*^*+/+*^ and *Robo4*^*−/−*^*;Robo4ΔCD* mouse serum. (**l**) sRobo4 expression analysis by ELISA with mouse serum from 6–8 week old mice. *N*=10–17 mice in each group. The data represent mean±s.e.m. *P<0.05; **P<0.01; ***P<0.001, Welch's *t*-test.

We used a mouse Robo4ΔCD cDNA construct (aa 1–522) that expressed the ECD and TM domain but lacked >95% of ICD sequence and was fused to GFP (Supplementary Fig. 1). The construct was placed under the control of a tetracycline-responsive element (TET-off system) and used to generate transgenic mice. These mice were crossed with a CDH5-promoter-driven tetracycline-transactivator (CDH5-tTA) transgenic mouse line^50^. The double transgenic mice (hereafter *Robo4*^*+/+*^*;Robo4ΔCD* mice) were expected to express CDH5-driven Robo4ΔCD in endothelial cells and additionally expressed endogenous Robo4. Mice carrying either of the single transgenes expressed endogenous Robo4 but not *Robo4ΔCD* and were used as controls (*Robo4*^*+/+*^*;Stg* mice) (Fig. 1a). We also generated mice expressing Robo4ΔCD in the absence of endogenous Robo4 (hereafter *Robo4*^*−/−*^*;Robo4ΔCD* mice), by intercrossing the double transgenic mice with *Robo4*^*−/−*^ mice^18^.

*Robo4*^*+/+*^*;Robo4ΔCD* and *Robo4*^*−/−*^*;Robo4ΔCD* mice were born at the expected Mendelian frequency in the absence of doxycycline treatment (Fig. 1b), indicating that neither lack of the full-length protein, nor lack of its cytoplasmic domain affected embryonic vascular development. Western blot analysis with an anti-Robo4 antibody on mouse lung endothelial cell (MLEC) protein extracts confirmed the presence of a single 140 kDa Robo4 band in wild-type mice, lack of expression in *Robo4*^*−/−*^ and *Robo4*^*−/−*^*;Stg* mice and expression of a shorter ∼100 kDa Robo4ΔCD form corresponding to the ECD (60 kDa), TM domain and GFP (together approximately 40 kDa) in cells isolated from *Robo4*^*−/−*^*;Robo4ΔCD* mice (Fig. 1c). Quantification of western blots showed that expression levels of Robo4ΔCD were similar to endogenous Robo4, indicating that transgenic mice expressed physiological levels of the mutant protein (Fig. 1d). Because it was GFP-tagged, the 100 kDa Robo4ΔCD protein could also be detected by western blot with an anti-GFP antibody in mouse lung lysates from *Robo4*^*+/+*^*;Robo4ΔCD* mice but not from *Robo4*^*+/+*^*;Stg* mice (Fig. 1e). Anti-GFP staining of *Robo4*^*+/+*^*;Robo4ΔCD* postnatal retina vasculature, adult aorta endothelium and primary MLECs showed specific transgene expression in endothelial cells (Fig. 1f–h). The Robo4ΔCD-GFP fusion protein was primarily localized on the cell surface, with enrichment in the lateral junctional areas (Fig. 1g,h). Expression of mCherry-tagged Robo4ΔCD construct in HUVECs confirmed cell surface localization of the mutant protein (Fig. 1i; Supplementary Fig. 2).

We also detected a soluble Robo4 (sRobo4) form of 60 kDa using immunoprecipitation with an anti-Robo4 antibody in human serum and in supernatant collected from human umbilical artery ECs (HUAECs, Fig. 1j). Based on its molecular weight, sRobo4 is likely to encode most of the ECD. Expression of Robo4-full length (FL) and ΔCD constructs in porcine aortic endothelial cells (PAECs) led to sRobo4 secretion into the supernatant, whereas untransfected PAECs did not express sRobo4 (Fig. 1j). sRobo4 was also detected in mouse serum from wild-type and *Robo4*^*−/−*^*;Robo4ΔCD* mice, but was absent in *Robo4*^*−/−*^ mice (Fig. 1k). ELISA analysis of serum confirmed the presence of sRobo4 in *Robo4*^*+/+*^*;Robo4ΔCD* mice but not in *Robo4*^*−/−*^ mice (Fig. 1l). The serum level of sRobo4 is ∼7 ng ml^−1^ in *Robo4*^*+/+*^ mice, ∼25 ng ml^−1^ in *Robo4*^*+/+*^*;Robo4ΔCD* and ∼20 ng ml^−1^ in *Robo4*^*−/−*^*;Robo4ΔCD* mice, demonstrating moderate overexpression of sRobo4 in transgenic mice (Fig. 1l).

### Robo4ΔCD inhibits OIR revascularization

To assess effects of Robo4ΔCD on developmental angiogenesis, we analysed postnatal retinas of Stg and Robo4ΔCD expressing mice on both wild-type and *Robo4* knockout background. We did not see any differences in vessel outgrowth and vessel branching between Robo4ΔCD expressing mice and their control littermates at P7 (Supplementary Fig. 3a).

We next challenged mice with OIR. After hyperoxia exposure, P12 *Robo4*^*+/+*^*, Robo4*^*−/−*^, *Robo4*^*−/−*^*;Stg* and *Robo4*^*−/−*^*;Robo4ΔCD* pups all developed comparable vaso-obliteration, leading to a capillary-free avascular area in the centre of the retina (Supplementary Fig. 3b,c). Hence, Robo4 did not affect hyperoxia-induced vaso-obliteration.

**Figure 2:**
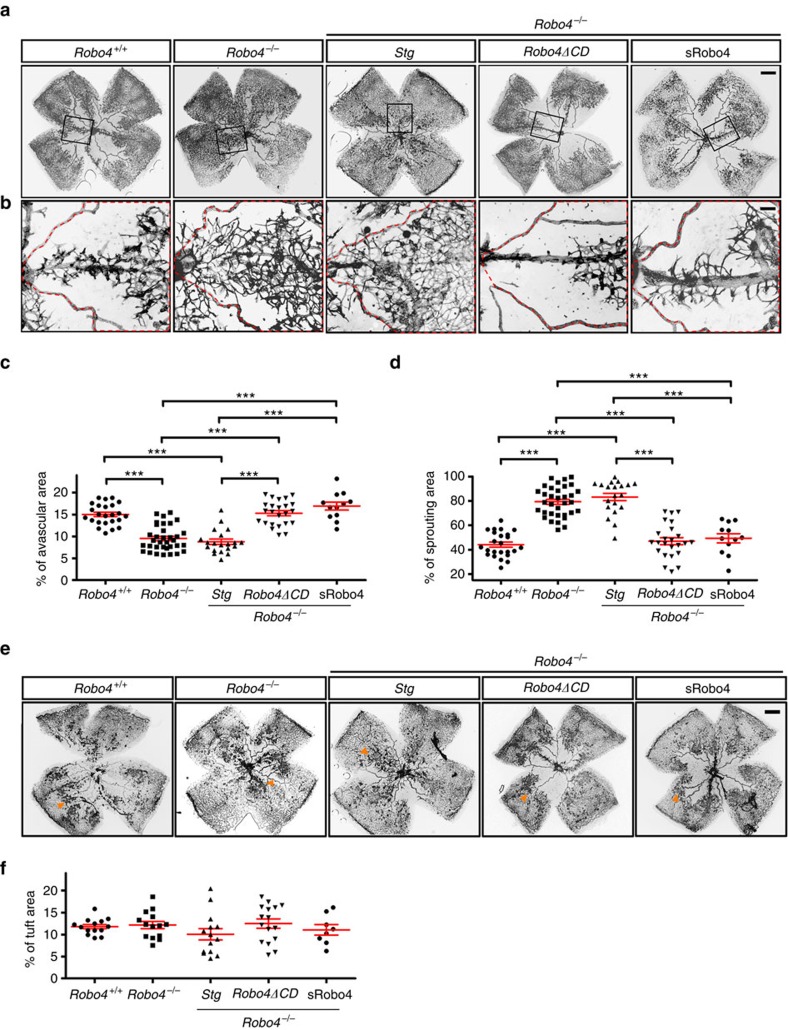
Robo4 inhibits OIR revascularization independently of its cytoplasmic domain. (**a**) IsoB4-stained whole-mount retinal vasculature of P17 mice with the indicated genotypes after OIR. SRobo4 was injected intraperitoneally daily from P12 to P16. Scale bar, 500 μm. (**b**) Higher magnification images of boxed areas in **a** showing vessels sprouting from veins. Scale bar, 100 μm. (**c**) Quantifications of avascular area in the whole retinas shown in **a**. (**d**) Quantifications of vessel coverage in the red stippled area in **b**. Each dot represents a retina. *N*=12–34 retinas (6–17 mice) per group. Error bars: s.e.m. ***P<0.001, Mann–Whitney *U* test. (**e**) IsoB4-stained neovessel tufts of P17 mice with the indicated genotypes after OIR. To preferentially stain neovascular tufts (arrowheads), retinas were subjected to mild detergent permeabilization and short IsoB4 incubation. Scale bar, 500 μm. (**f**) Quantifications of tuft area in the retinas shown in **e**. *N*=8–16 retinas (4–8 mice) per group. Error bars: s.e.m. No significant differences in tuft area were observed using Mann–Whitney *U* test.

After return to room air, hypoxia in the avascular area triggered re-growth of normal vessel sprouts from centrally located veins and the remaining capillaries in the periphery (Fig. 2a,b), and pre-retinal neovascular tufts (Fig. 2e, see arrowheads for tufts). Compared with *Robo4*^*+/+*^, *Robo4*^*−/−*^ mice showed increased revascularization, characterized by a significant decrease of the retina avascular area, and increased sprouting from veins (Fig. 2a–d). Expression of Robo4ΔCD in *Robo4*^*−/−*^ mice prevented the increased revascularization (Fig. 2a–d), indicating that Robo4ΔCD rescued Robo4 function during ocular revascularization. Intraperitoneal injection of recombinant sRobo4 protein encoding His-tagged ECD^18^ in *Robo4*^*−/−*^ mice inhibited OIR revascularization (Fig. 2a–d), demonstrating that sRobo4 is sufficient to activate signalling. Interestingly, neovascular tuft formation was unchanged in *Robo4*^*−/−*^ and *Robo4*^*−/−*^;*Robo4ΔCD* mice and after sRobo4 injection (Fig. 2e,f), indicating that Robo4 controlled revascularization but was dispensable for pathological neovascular tuft formation in the OIR model.

**Figure 3:**
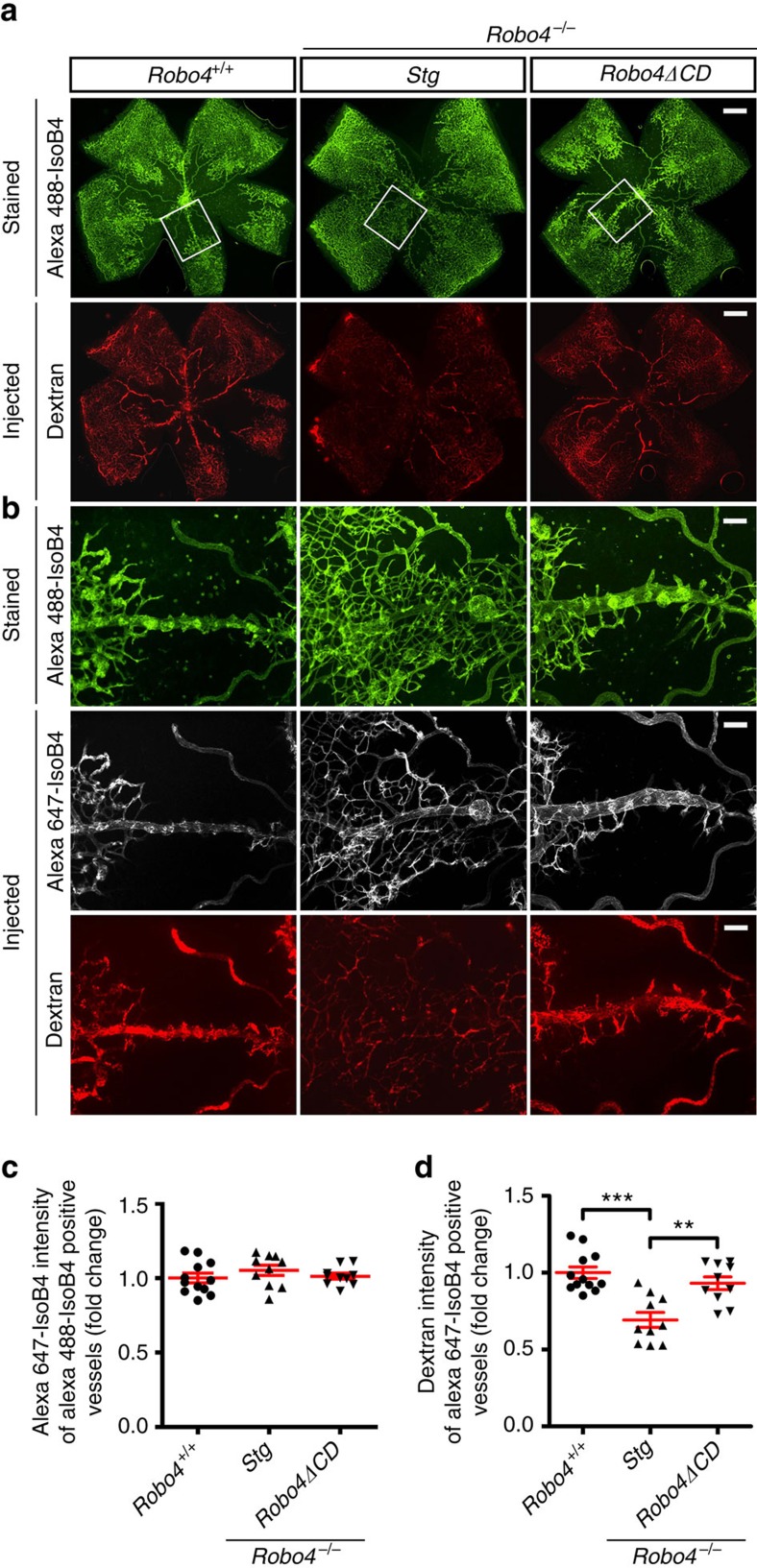
Robo4ΔCD inhibits OIR retinal vessel leakage. P17 OIR mice of the indicated genotypes were injected with Rhodamine-dextran and Alexa 647-IsoB4 for 5 min, then mice were sacrificed and vasculature was stained with Alexa 488-IsoB4. (**a**) Low-magnification images of the Alexa 488-IsoB4-stained vasculature (upper panel, green) and Rhodamin-dextran perfusion (red, lower panel). Note reduced intensity of dextran labelling indicating increased permeability in *Robo4*^*−/−*^*;Stg* mice compared with *Robo4*^*+/+*^ and *Robo4*^*−/−*^*;Robo4ΔCD* mice. Scale bars, 500 μm. (**b**) Higher magnification images of the boxed areas in **a**. Upper panel: Alexa 488-IsoB4-stained vasculature. Middle panel: injected Alexa 647-IsoB4-labelled vasculature. Lower panel: injected rhodamine-dextran labelled vasculature. Scale bars, 100 μm. (**c**) Quantifications of injected Alexa 647-IsoB4 fluorescence intensity in Alexa 488-IsoB4-labelled vasculature in images shown in **b**. Each dot represents a retina. *N*=10–12 retinas (5–6 mice) per group. Error bars represent s.e.m. Note no significant difference between genotypes, indicating equal perfusion of all groups. (**d**) Quantification of dextran fluorescence intensity in Alexa 647-IsoB4-labelled vasculature. Data were normalized to Alexa 647-IsoB4 intensity and are presented as fold change compared with *Robo4*^*+/+*^ mice. *Robo4*^*−/−*^*;Stg* mice show reduced intensity of dextran labelling hence increased leakage, this is rescued in *Robo4*^*−/−*^*;Robo4ΔCD* mice. Each dot represents a retina. *N*=10–12 retinas (5–6 mice) per group. Error bars represent s.e.m. **P<0.01; ***P<0.001, Mann–Whitney *U* test.

To examine vascular leak, we injected P17 OIR *Robo4*^*+/+*^, *Robo4*^*−/−*^*;Stg* and *Robo4*^*−/−*^;*Robo4ΔCD* mice retro-orbitally with 70 kDa rhodamine-dextran and fluorescent Alexa 647 conjugated IsoB4, which labelled the luminal endothelial membrane of perfused vessels. Dyes were left to circulate for 5 min, then retinas were harvested and re-stained with Alexa 488 conjugated IsoB4 (Fig. 3a,b). Overlay of injected and stained IsoB4 revealed efficient perfusion in all genotypes (Fig. 3a–c). *Robo4*^*−/−*^*;Stg* mice showed increased vessel leak, as attested by reduced dextran labelling of Alexa 647 IsoB4+ retinal vasculature compared with wild-type mice (Fig. 3b,d). Dextran labelling of *Robo4*^*−/−*^;*Robo4ΔCD* retinas was similar to that seen in wild-type mice, demonstrating that *Robo4ΔCD* was sufficient to rescue vascular leak (Fig. 3b,d).

### Robo4ΔCD inhibits vascular leak and wound healing

**Figure 4:**
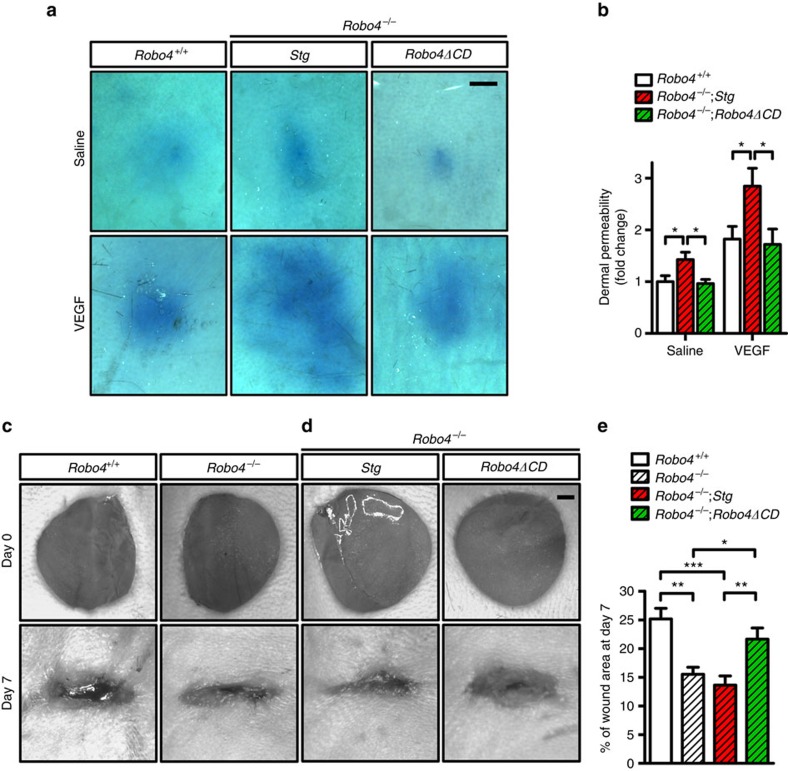
Robo4ΔCD inhibits dermal vascular permeability and wound healing. (**a**) Miles dermal permeability analysis. Evans blue leakage was assessed 30 min after intradermal injection of saline or 50 ng VEGF into the indicated mice. Animals were PBS perfused before imaging and quantification. Note increased leak in VEGF treated *Robo4*^*−/−*^*;Stg* compared with *Robo4*^*+/+*^ and *Robo4*^*−/−*^*;Robo4ΔCD* mice. Scale bar, 2 mm. (**b**) Quantification of Evans blue dye in the skin. *N*= 5–8 mice per group. Error bars represent s.e.m. *P<0.05, Mann-Whitney *U* test. (**c**,**d**) Representative images of wound healing of mice with the indicated genotypes at day 0 and 7 after cutaneous punch biopsy. Scale bar, 1 mm. (**e**) Quantification of percentage of wound area at day 7 after wounding. *N*=14 wounds (7 mice) in each group. Error bars: s.e.m. *P<0.05; **P<0.01; ***P<0.001, Mann–Whitney *U* test.

As *Robo4*^*−/−*^ mice exhibit enhanced VEGF-driven dermal vessel permeability^17^,^18^, we tested effects of Robo4ΔCD in a Miles assay. To this end, Evans blue was injected intravenously followed by an intradermal injection of saline or VEGF into *Robo4*^*+/+*^, *Robo4*^*−/−*^*;Stg* and *Robo4*^*−/−*^;*Robo4ΔCD* mice. After 30 min, animals were sacrificed, perfused with PBS and extravasated dye was imaged and quantified. With saline injection, *Robo4*^*−/−*^*;Stg* mice exhibited slightly increased leakage of the Evans blue dye into the skin when compared with *Robo4*^*+/+*^ and *Robo4*^*−/−*^;*Robo4ΔCD* mice (Fig. 4a,b). Injection of VEGF into the control wild-type mice led to a robust leakage of Evans Blue (Fig. 4a,b). *Robo4*^*−/−*^*;Stg* exhibited significantly increased leakage of the Evans blue dye into the skin (Fig. 4a,b). In contrast, VEGF injection caused much less dye leakage in *Robo4*^*−/−*^;*Robo4ΔCD* mice (Fig. 4a,b). To test whether this effect was specific for the VEGF pathway, we injected another potent inducer of vessel permeability, histamine. Histamine-induced permeability was similar in *Robo4*^+/+^;*Robo4ΔCD* and in *Robo4*^+/+^;*Stg* mice (Supplementary Fig. 4), suggesting that Robo4ΔCD specifically targets VEGF-induced permeability signalling and is sufficient to prevent this process.

To determine the functional outcome of enhanced revascularization and vessel permeability in *Robo4*^*−/−*^ mice, we next studied cutaneous wound healing. *Robo4*^*−/−*^ mice and *Robo4*^*−/−*^*;Stg* mice exhibited significantly faster wound closure than wild-type littermates at 7 days after wounding (Fig. 4c–e). Expression of Robo4ΔCD in *Robo4-null* background was sufficient to prevent accelerated wound healing (Fig. 4c–e).

### ROBO4ΔCD decreases VEGFR2 Y951 phosphorylation

**Figure 5:**
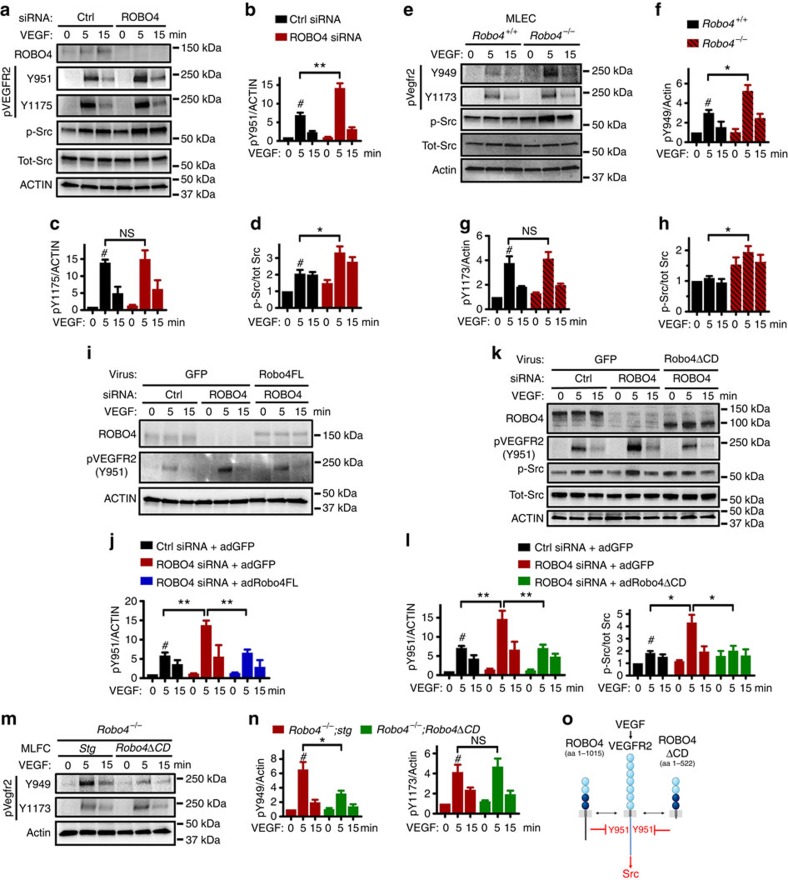
Robo4ΔCD affects VEGF signalling through VEGFR2 Y951. (**a**) Western blot analysis of VEGFR2 and Src phosphorylation in HUVECs transfected with Ctrl or *ROBO4* siRNAs and treated with 3 nM VEGF. (**b**–**d**) Quantifications of blots shown in **a**. #: VEGF significantly induces VEGFR2 pY951, pY1175 and p-Src at 5 min. *ROBO4* siRNA further increases pY951 and p-Src, but not pY1175. *N*=4–6 experiments. Error bars: s.e.m. NS, not significant; *P<0.05; **P<0.01, Student's *t*-test. (**e**) Western blot analysis of pVegfr2 and p-Src in MLECs from *Robo4*^*+/+*^ and *Robo4*^*−/−*^ mice after 6 nM VEGF stimulation. (**f**–**h**) Quantifications of blots shown in **e**. #: VEGF significantly induces Vegfr2 pY949 and pY1173. *Robo4*^*−/−*^ cells show further enhanced pY949 and p-Src, but not pY1173. *N*=3 experiments. Error bars: s.e.m. NS, not significant; *P<0.05, Student's *t*-test. (**i**) Western blot and (**j**) quantification of pY951 in HUVECs with indicated siRNA transfection and adenovirus infection after 3 nM VEGF stimulation. #: VEGF significantly induces VEGFR2 pY951 at 5 min. ROBO4FL rescues increased pY951 in *ROBO4* silenced cells. *N*=4 experiments. Error bars: s.e.m. **P<0.01, Student's *t*-test. (**k**) Western blot analysis of pY951 (*n*=6) and p-Src (*n*=4) and the corresponding quantifications (**l**) in HUVECs with indicated siRNA transfection and adenovirus infection after 3 nM VEGF stimulation. #: VEGF significantly induces VEGFR2 pY951 and p-Src at 5 min. Robo4ΔCD restores pY951 and p-Src in *ROBO4* silenced cells to Ctrl levels. Error bars: s.e.m. *P<0.05, **P<0.01, Student's *t*-test. (**m**) Western blot and (**n**) quantification of pVegfr2 in MLECs from *Robo4*^*−/−*^*;Stg* and *Robo4*^*−/−*^*;Robo4ΔCD* mice after 6 nM VEGF stimulation. Note decreased Y949 but unchanged Y1173 in *Robo4*^*−/−*^*;Robo4ΔCD. N*=5 experiments. Error bars: s.e.m. NS, not significant; *P<0.05, Student's *t*-test. (**o**) Schematic of ROBO4 and Robo4ΔCD effects on VEGF signalling through VEGFR2.

To address ROBO4 effects on VEGF signalling, we knocked down *ROBO4* in HUVECs with siRNA (Supplementary Table 1), which strongly decreased ROBO4 protein levels (Fig. 5a). *ROBO4* silencing accelerated VEGF-induced HUVEC monolayer permeability (Supplementary Fig. 5a), wound closure (Supplementary Fig. 5b,c) and increased vascularization in three-dimensional (3D) fibrin gels (Supplementary Fig. 5d,e).

To determine the mechanism responsible for the enhanced VEGF response, we treated control and *ROBO4* siRNA knockdown cells with VEGF for 5 and 15 min and examined phosphorylation of the VEGFR2 intracellular tyrosine residues Y951, Y1175 and Y1214. VEGF induced a significant increase of phosphorylation at all sites in control siRNA treated cells (Fig. 5a; Supplementary Fig. 6a). *ROBO4* knockdown further enhanced phosphorylation on Y951, but not on Y1175 or Y1214 (Fig. 5a–c; Supplementary Fig. 6a). Phosphorylated VEGFR2 Y951 activates c-Src^23^,^24^, and c-Src phosphorylation levels were also increased in *ROBO4* knockdown cells (Fig. 5a,d). Likewise, VEGF treatment of primary MLECs showed enhanced activation of Vegfr2 pY949 and Src phosphorylation in cells isolated from *Robo4*^−/−^ mice compared with *Robo4*^+/+^ mice, while Y1173 activation was similar between genotypes (Fig. 5e–h).

Treatment of *ROBO4* knockdown HUVECs with recombinant sRobo4 was sufficient to reduce VEGF-induced Y951 activation, suggesting that the ROBO4 cytoplasmic domain was dispensable for effects on VEGF signalling (Supplementary Fig. 6b). To test this further, we re-expressed siRNA-resistant full-length mouse Robo4 in *ROBO4* knockdown HUVECs, which prevented the increase in VEGF-induced Y951 phosphorylation (Fig. 5i,j; Supplementary Table 1). Next, we expressed siRNA-resistant mouse Robo4ΔCD in *ROBO4* knockdown HUVECs, which was sufficient to prevent the excessive pY951 and p-Src in response to VEGF (Fig. 5k,l; Supplementary Table 1). Consistently, both constructs also inhibited increased HUVEC monolayer permeability, wound closure and fibrin gel sprouting angiogenesis seen with VEGF treated, *ROBO4*-silenced cells (Supplementary Fig. 5). In primary MLECs, we observed that VEGF-induced Y949 and Src phosphorylation are reduced in *Robo4*^*−/−*^*;Robo4ΔCD* mice compared with *Robo4*^*−/−*^*;Stg* littermates, while Y1173 activation is similar between genotypes (Fig. 5m,n; Supplementary Fig. 6c). Thus, ROBO4 selectively targets the VEGFR2 Y951/949-Src signalling cascade independently of its cytoplasmic domain (Fig. 5o).

### ROBO4 is dispensable for Slit2 signalling

**Figure 6:**
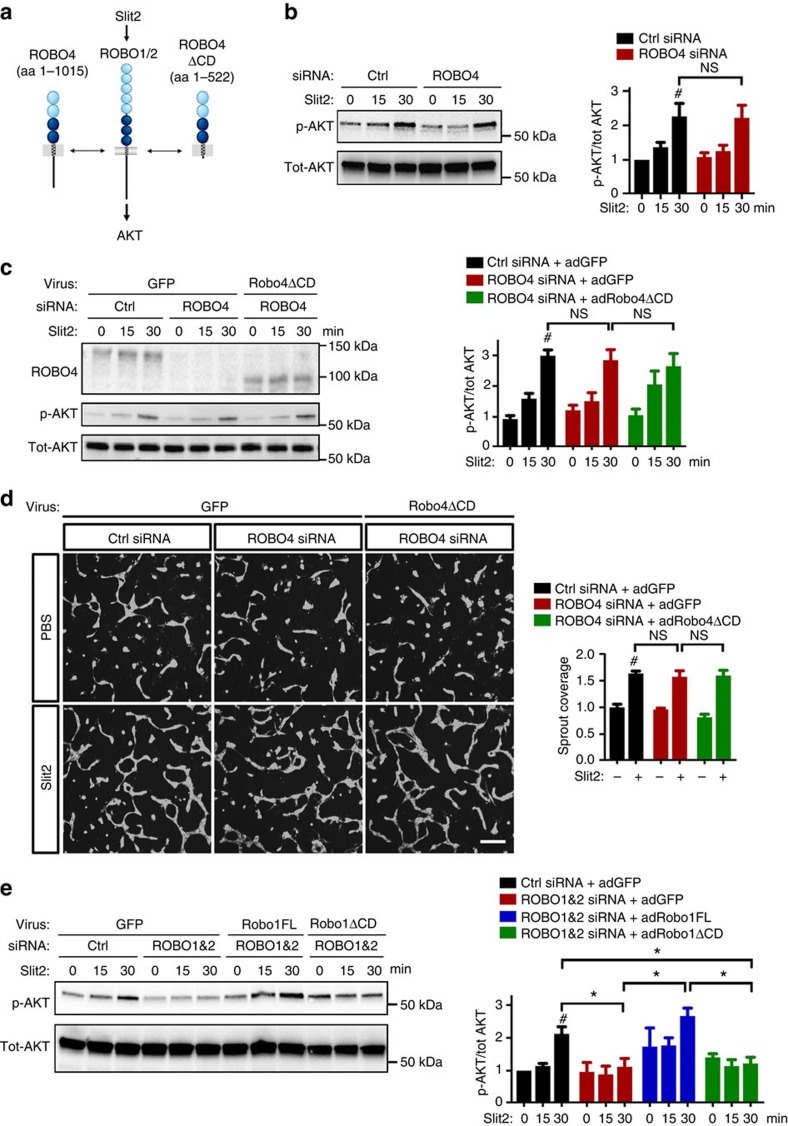
ROBO4ΔCD does not affect Slit2-mediated AKT activation and sprouting. (**a**) Schematic of possible ROBO4 and Robo4ΔCD effects on Slit2-ROBO1/2 signalling. (**b**) Western blot of phospho-AKT S473 (p-AKT) (left) and quantification (right) in HUVECs with Ctrl or *ROBO4* siRNA transfection and 6 nM Slit2 stimulation. #: Slit2 significantly induces p-AKT at 30 min. *N*=3 experiments. Error bars: s.e.m. NS, not significant, Student's *t*-test. (**c**) Left panel: western blot analysis of ROBO4 expression and p-AKT in HUVECs with indicated siRNA transfection and adenovirus infection after 6 nM Slit2 stimulation. Right panel: western blot quantifications of p-AKT/tot-AKT (*n*=4). #: Slit2 significantly induces p-AKT at 30 min. Error bars: s.e.m. NS, not significant, Student's *t*-test. (**d**) HUVEC sprouting in 3D fibrin gels (left) and the corresponding quantifications (right, *n*=3). Cells were treated with siRNAs and virus as indicated and then stimulated with 6 nM Slit2 for 96 h. #: Slit2 significantly induces HUVEC sprouting. Error bars: s.e.m. NS, not significant, Mann–Whitney *U* test. Scale bar, 200 μm. (**e**) Western blot analysis of p-AKT in HUVECs with indicated siRNA transfection and adenovirus infection after 6 nM Slit2 stimulation. *N*=3 experiments. #: Slit2 significantly induces p-AKT at 30 min. Error bars: s.e.m. * *P*<0.05 when compared with Ctrl siRNA+adGFP group, Student's *t*-test.

To determine if enhanced angiogenesis in the absence of Robo4 could be due to dominant negative effects on Slit2 signalling through Robo1, we tested if ROBO4 affected Slit2 signalling in HUVECs *in vitro* (Fig. 6a). *ROBO4* siRNA knockdown in HUVECs did not affect mRNA expression levels of ROBO1 and 2 (Supplementary Fig. 7). Stimulation with recombinant Slit2 and western blot analysis showed that Slit2 induced AKT phosphorylation, and that *ROBO4* silencing did not affect the level of Slit2-induced p-AKT (Fig. 6b). Expression of Robo4ΔCD in *ROBO4* siRNA knockdown cells did not affect Slit2-induced AKT activation (Fig. 6c). We next tested effects of ROBO4 on Slit2-induced angiogenic sprouting in 3D fibrin gels. Again, neither *ROBO4* siRNA, nor Robo4ΔCD affected sprouting induced by Slit2 (Fig. 6d). In contrast to ROBO4, silencing of *ROBO1* and *2* abolished Slit2-induced AKT activation (Fig. 6e). Reconstitution of *ROBO1* and *2* knockdown cells with adenovirus encoding full-length siRNA-resistant rat Robo1 rescued Slit2-induced AKT activation (Fig. 6e; Supplementary Table 1). AKT activation of *ROBO1* and *2* knockdown cells was not rescued by expression of Robo1ΔCD (Fig. 6e), indicating that the Robo1 ICD is required for Slit2 signalling while ROBO4 does not interfere with Slit2 mediated AKT activation and sprouting.

### ROBO4 affects VEGFR2 pY951 via UNC5B

**Figure 7:**
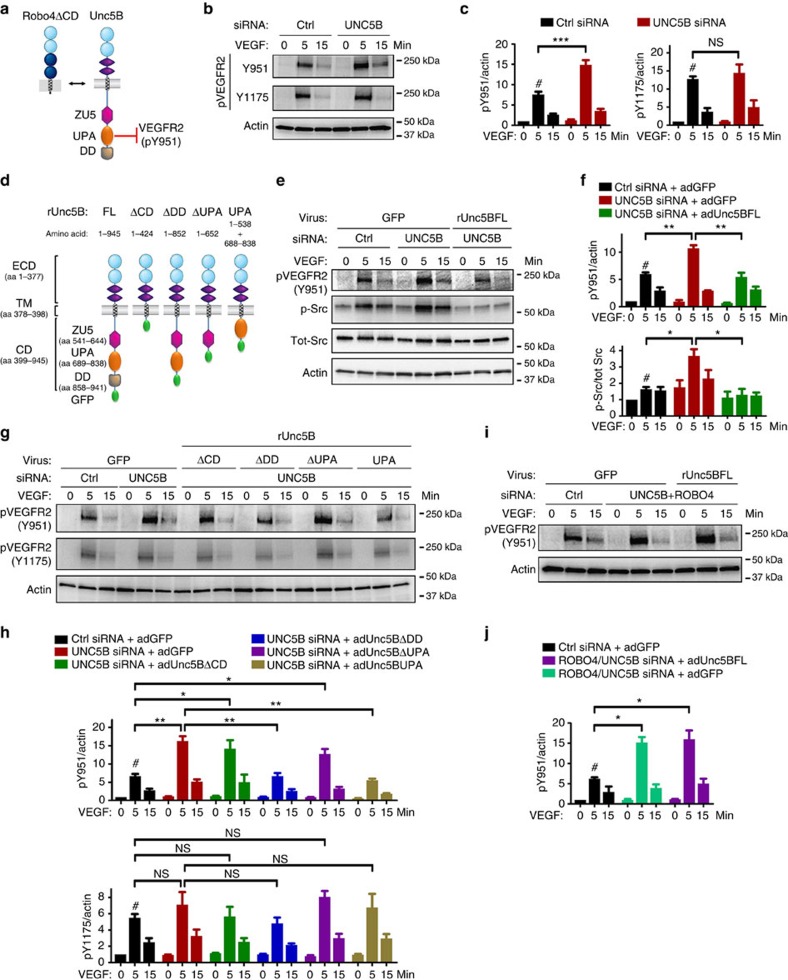
ROBO4 regulates VEGF signalling through UNC5B. (**a**) Proposed model for ROBO4 inhibition of VEGFR2 Y951-Src phosphorylation via the UNC5B UPA domain. (**b**) Western blot analysis of VEGFR2 pY951 and pY1175 in HUVECs transfected with Ctrl or *UNC5B* siRNA and treated with 3 nM VEGF. (**c**) Quantifications of blots shown in **b**. #: VEGF significantly induces pY951 and pY1175 at 5 min. *UNC5B* siRNA further enhances pY951 but not pY1175. *N*=5 experiments, Error bars: s.e.m. ***P<0.001, Student's *t*-test. (**d**) Schematic diagram showing the engineering of various CD-truncated UNC5B mutants. (**e**) Western blot analysis and (**f**) quantifications of pY951 and p-Src in HUVECs with indicated siRNA transfection and adenovirus infection after 3 nM VEGF stimulation. #: VEGF significantly induces pY951 and p-Src at 5 min. Unc5BFL rescues increased pY951 and p-Src in *UNC5B* silenced cells. *N*=3 experiments. Error bars: s.e.m. *P<0.05 **P<0.01, Student's *t*-test. (**g**) Western blot analysis and (**h**) quantification of pVEGFR2 in HUVECs with indicated siRNA transfection and adenovirus infection after 3 nM VEGF stimulation. #: VEGF significantly induces pY951 and pY1175 at 5 min. Unc5BΔDD and Unc5BUPA but not Unc5BΔCD and Unc5BΔUPA rescue the excessive VEGFR2 Y951 phosphorylation in *UNC5B* knockdown cells. None of the constructs affect Y1175 phosphorylation. *N*=3 experiments. Error bars: s.e.m. *P<0.05; **P<0.01, Student's *t*-test. (**i**) Western blot analysis and (**j**) quantification of pY951 (*n*=3) in HUVECs with indicated siRNA transfection and adenovirus infection after 3 nM VEGF stimulation. #: VEGF significantly induces pY951 at 5 min. Note that expression of Unc5BFL could not rescue the excessive VEGFR2 Y951 phosphorylation in *UNC5B* and *ROBO4* double siRNA-transfected cells. Error bars: s.e.m. *P<0.05, Welch's *t*-test.

As ROBO4 binds to UNC5B and activates UNC5B signalling^18^, we next examined the effect of UNC5B on VEGF-induced pY951 (Fig. 7a). We knocked down *UNC5B* in HUVECs with siRNA (Supplementary Table 1), which strongly decreased *UNC5B* mRNA levels (Supplementary Fig. 7b). We found that *UNC5B* silencing also enhanced VEGF-induced VEGFR2 pY951 without affecting phosphorylation of the Y1175 residue (Fig. 7b,c). Re-expression of siRNA-resistant rat full-length Unc5B (rUnc5BFL, Fig. 7d; Supplementary Fig. 7c) in *UNC5B*-silenced cells rescued VEGF-induced VEGFR2 pY951 and Src phosphorylation (Fig. 7e,f). These data suggested that ROBO4 could affect VEGF signalling via UNC5B.

To understand which domains of UNC5B were required for VEGF signalling, we generated various Unc5B cytoplasmic domain deletions (Fig. 7d). Expression of a rat Unc5B construct deleted in its entire cytoplasmic domain (rUnc5BΔCD) in *UNC5B* knockdown cells failed to rescue VEGF-induced VEGFR2 pY951 phosphorylation (Fig. 7g,h; Supplementary Fig. 7c), suggesting that the Unc5B signalling domain was required to modulate VEGFR2 activation. The Unc5B cytoplasmic domain contains a death domain (DD) implicated in cell survival, a UPA domain (abbreviated for conserved in *U*nc5B, *P*idd and *A*nkyrin) and a ZU5 domain^51^ (Fig. 7d). Expression of rUnc5BΔDD lacking the death domain in *UNC5B* knockdown cells rescued VEGF-induced VEGFR2 pY951 phosphorylation (Fig. 7g,h; Supplementary Fig. 7c), indicating that the DD is dispensable for Unc5B effects on VEGFR2 signalling. In contrast, expression of rUnc5BΔUPA in *UNC5B* knockdown cells failed to rescue VEGF-induced VEGFR2 pY951 phosphorylation (Fig. 7g,h; Supplementary Fig. 7c), indicating that the UPA domain contains residues required for modulation of VEGFR2 signalling. To test if the UPA domain was sufficient to target VEGFR2 Y951, we generated a construct containing only the cytoplasmic rUnc5B UPA domain linked to Unc5B transmembrane and extracellular domain (Fig. 7d). Expression of this construct in *UNC5B* knockdown cells was sufficient to rescue VEGFR2 Y951 activation (Fig. 7g,h; Supplementary Fig. 7c). None of the Unc5B constructs affected VEGFR2 Y1175 phosphorylation, demonstrating a highly specific effect of the activated UPA domain on Y951 (Fig. 7g,h; Supplementary Fig. 7c). Finally, expression of rUnc5BFL in cells silenced for both *UNC5B* and *ROBO4* failed to rescue Y951 activation (Fig. 7i,j), demonstrating that UNC5B cannot modulate VEGFR2 in the absence of ROBO4. For uncropped versions of all immunoblots shown in this study, see Supplementary Fig. 8.

These data support a model in which ROBO4 decreases VEGFR2 Y951 activation by binding to UNC5B and activating signalling through the UNC5B UPA domain (Fig. 7a).

## Discussion

We show here that Robo4 deletion enhances revascularization in OIR models and during cutaneous wound healing. Transgenic expression of near-physiological levels of Robo4ΔCD suppresses VEGF-induced vessel permeability, excessive angiogenesis and would healing in *Robo4*^*−/−*^ mutants. These results demonstrate that Robo4 restrains angiogenesis independently of its cytoplasmic domain.

The OIR retina undergoes two types of angiogenesis: revascularization and neovascular tuft formation^20^,^21^. Revascularization is considered as physiological ‘healing' of the injured retina and leads to formation of normal vasculature that eliminates ischemia, while pathological neovascularization results in formation of fragile balloon-like vessels prone to bleeding, hence aggravating ischemic tissue injury^20^,^21^. Our results show that Robo4 blockade enhances OIR revascularization and vessel permeability without exacerbating pathological tuft formation. Thus, blocking ROBO4 might be beneficial to accelerate revascularization in ROP. Likewise, Robo4 blocking accelerates wound closure in endothelial monolayers and promotes cutaneous wound healing, suggesting that ROBO4 blockade could be used to promote wound healing in diabetic patients.

The data are inconsistent with a model where Robo4 signals through its ICD to suppress angiogenesis. Thus, even though the Robo4 ICD can interact with various cytoplasmic signalling molecules^2^,^26^,^34^,^35^, this association appears dispensable for the *in vivo* Robo4 effects described in this study. Immunolocalization studies in HUVECs had previously shown that full-length ROBO4 shuttles between the plasma membrane and intracellular vesicles, while Robo4ΔCD is readily detectable at the plasma membrane^18^,^35^. We speculate that the ROBO4 ICD may control its trafficking between intracellular vesicles and plasma membrane, perhaps via association with some of the previously identified binding partners, many of which are implicated in endocytosis and vesicle trafficking^52^,^53^,^54^,^55^.The molecular details of this remain to be elucidated, but appear of minor relevance for ROBO4 biological function.

Interestingly, we find that a soluble 60 kDa form of Robo4 is detectable in supernatant from Robo4 expressing cells and in mouse and human serum. The size of sRobo4 suggests that it is a proteolytic fragment that contains most of the ECD, including the N-terminal Ig-like domains that bind UNC5B (see below)^18^. Thus, sRobo4 appears to be endowed with biological activity and its release into the circulation could activate Robo4 signalling to inhibit VEGF-induced permeability and angiogenesis. Increased plasma Robo4 levels in patients with tumours or vascular injury^3^,^56^ may therefore reflect a physiological strategy to protect vasculature from hyperpermeability and excessive angiogenesis. Alternatively, sRobo4 could interfere with the interaction between membrane-bound Robo4 and UNC5B and inhibit UNC5B signalling.

Having established that the ICD is dispensable for ROBO4 function, we tested if ROBO4 restrained angiogenesis by heterodimerization with ROBO1. MLECs and HUVECs express 10-fold higher levels of ROBO4 than of ROBO1 (ref. 32), and both receptors can be co-immunoprecipitated from endothelial cell lysates^35^, suggesting that ROBO4 could affect Slit2 signalling through ROBO1. Since ROBO4 lacks Slit2 binding residues^36^,^37^,^38^,^39^, its heterodimerization with ROBO1 could lead to formation of complexes unable to bind and transduce Slit2 signals. Inducible loss of *Slit2* or combined deletion of *Robo1* and *2* function leads to severe angiogenic sprouting defects in OIR models^32^, while *Robo4* deletion leads to enhanced sprouting angiogenesis (Fig. 2), suggesting that Robo4 could restrain ocular vascularization by blocking Slit2 signalling through Robo1 and 2. However, in contrast to Slit2 mutant mice, neither *Robo4*^*−/−*^ nor *Robo1*^*−/−*^*;Robo4*^*−/−*^ showed any developmental angiogenesis defects^32^. Moreover, our signalling experiments in HUVECs failed to reveal any effect of *ROBO4* knockdown or Robo4ΔCD expression on Slit2 signalling. Instead, we find that Slit2 activation of downstream AKT can be completely blocked by *ROBO1* and *2* knockdowns or ROBO1ΔCD expression. These data are fully consistent with genetic evidence from studies with *Slit2* and *Robo1* and *2* receptor loss-of-function mutants^32^ and reveal opposing actions of Robo1, 2 and 4 in ocular vascularization *in vivo*: Slit2 signals through Robo1 and 2 to promote OIR angiogenesis, while Robo4 restrains this process independently of its cytoplasmic domain. Thus, heterodimerization of ROBO1 and 4 does not appear to play a biological role in the context of angiogenesis *in vivo*.

Although ROBO4 failed to show effects on Slit2 signalling, it readily affected VEGF signalling by targeting a specific VEGFR2 intracellular tyrosine, Y951, which controls VEGF-induced c-Src activation, vascular permeability and pathological angiogenesis^22^,^23^,^24^,^25^. Since *ROBO4* knockdown enhanced VEGF-induced VEGFR2 Y951 and downstream Src phosphorylation, ROBO4 appears to function as a selective inhibitor of this site's activation. By contrast, ROBO4 fails to affect other intracellular VEGFR2 phosphosites including Y1175, which is critical for PLC-γ and downstream ERK activation^57^. Exchange of the Y1173 residue in mouse VEGFR2 (Y1175 in human VEGFR2) for phenylalanine results in arrested endothelial cell development and embryonic death, similar to the global *Vegfr2*^*−/−*^ phenotype^58^. Selective targeting of Y951 but not Y1175 by ROBO4 is consistent with the phenotype of *Robo4*^*−/−*^ mice and may explain why these mice are deficient in angiogenesis under pathological condition, but not angiogenesis during development.

Expression of Robo4ΔCD was sufficient to rescue VEGF-induced Y951/Y949 activation and Src phosphorylation *in vitro* and *in vivo*, demonstrating that the ROBO4 ICD is dispensable for its effects on VEGF signalling. We had previously shown that the ROBO4 ECD binds to the ECD of another endothelial guidance receptor UNC5B^18^, prompting us to investigate if ROBO4 could signal through UNC5B to counteract VEGFR2 Y951 activation. Indeed, we find that like *ROBO4* knockdown, *UNC5B* knockdown enhances Y951 but not Y1175 phosphorylation in response to VEGF and that re-expression of Unc5B-FL rescues Y951 activation in *UNC5B* but not in *ROBO4* knockdown cells, supporting a function of UNC5B downstream of ROBO4. Structure–function analysis showed that effects on Y951 depend on the UPA domain in the UNC5B ICD, together supporting a model where ROBO4 affects Y951 by binding and signalling through UNC5B UPA.

How UPA signals remains to be clarified. The domain organization pattern of the UNC5 ICD (that is, ZU5-UPA-DD) is also found in ankyrins, scaffolding proteins regulating the assembly of specialized membrane microdomains^59^, and PIDD, scaffolding proteins controlling programmed cell death^60^,^61^,^62^. Crystal structure analysis has shown that when engaged with ligand (such as endothelial ROBO4), the UNC5B ZU5 and DD domains adopt a closed conformation, leaving the UPA domain exposed and thus able to interact with cytoplasmic signalling molecules^51^. UPA contains several tyrosines that might constitute docking sites for kinases or phosphatases regulating VEGFR2 Y951, but the molecular details remain to be established. Of note, our demonstration that UPA is sufficient to mediate ROBO4-UNCB5 effects on Y951 indicate a critical role for this domain and allow focusing future experiments on this domain.

In addition to Robo4, Unc5B binds to various other secreted ligands and transmembrane molecules including Netrins, BMP family members, Neogenin and the fibronectin and leucine-rich transmembrane protein Flrt3 (refs 63, 64, 65, 66, 67). Of these, Flrt3 is also expressed in endothelial cells, and conditional *Flrt3* knockout mice develop developmental retinal hypervascularization similar to that seen in Unc5B-mutant mice^18^,^68^. Thus, in contrast to Robo4 mutants, which show normal developmental angiogenesis, both Flrt3 and Unc5B mutants show impaired developmental angiogenesis, suggesting that additional signalling pathways relevant to vascular development are targeted by this interaction. While full elucidation of Unc5B signalling is beyond the scope of the current work, the data shown here demonstrate that endothelial ROBO4 targets VEGFR2 Y951 via the UNC5B UPA domain, thereby counteracting Y951-mediated vessel permeability and angiogenesis. Selectively enhancing VEGFR2 Y951 activation by blocking ROBO4 function targets revascularization without affecting neovascular tuft formation. The reason for this differential behaviour might be because tuft formation involves proliferation, which is not regulated by Y951 but via the VEGFR2 1175 residue. Taken together, we propose that Robo4 blockade might represent an opportunity to accelerate revascularization in retinopathy of prematurity and wound healing in diabetic patients.

## Methods

### Mice

The Yale University Institutional Animal Care and Use Committee (IACUC) reviewed and approved all mouse experiments.

Transgenic mice were generated at Yale Animal Genomics Services Centre. A mouse Robo4 fragment lacking >95% of the cytoplasmic domain (aa 1–522) was fused with GFP (Supplementary Fig. 1) and inserted 3' to the tetracycline-responsive element (TRE)-CMV promoter of the TET-off vector (Clontech). The resulting vector was linearized, purified and microinjected into mouse donor zygotes that were implanted into pseudo-pregnant mice. The resulting founder TET-Robo4ΔCD-GFP transgenic mice (C57BL/6J X SJL/J) were propagated on a C57BL/6J background and intercrossed with Cdh5-tTA transgenic mice^50^. Genotyping was performed by detecting the GFP-tag, and the Cdh5-tTA transgene (Supplementary Table 2)^52^. *Robo4^−/−^* mice on a C57BL/6J background were described previously^18^. To express Robo4ΔCD without endogenous Robo4, *Cdh5-tTA;Robo4ΔCD* transgenic mice were intercrossed with *Robo4^−/−^* mice.

### Antibodies and reagents

The detailed information of all antibodies used in this study is listed in Supplementary Table 3.

Recombinant VEGF-A165 (#293-VE) and Slit2 (#5444-SL) were from R&D Systems (Minneapolis, MN). The AllStars negative control siRNA (#SI03650318) was from Qiagen (Valencia, CA). Human ROBO1 (SMARTpool: ON-TARGETplus ROBO1 siRNA, #L-011381-00), ROBO2 (SMARTpool: ONTARGETplus ROBO2 siRNA, #L-023273-01), ROBO4 (SMARTpool: ON-TARGETplus ROBO4 siRNA, #L-015216-01) and UNC5B (SMARTpool: ON-TARGETplus UNC5B siRNA, #L-021392-00) siRNAs (Supplementary Table 1) were from Thermo Scientific (Waltham, MA). Anti-mouse, anti-rabbit and anti-goat secondary Abs conjugated to horseradish peroxidase (HRP) were from Vector Laboratories (Burlingame, CA). Sheep anti-rat IgG conjugated Dynabeads (#11035), lysine fixable rhodamine dextran (70 kDa MW, #D1818) and Isolectin B4 (IsoB4) conjugated to Alexa Fluor 488, 546 or 647 were from Invitrogen (Grand Island, NY). Human fibrinogen (#F8630), heparin (#H3149), thrombin (#T9549) and histamine (#H7125) were from Sigma-Aldrich (St Louis, MO).

### Cell culture and cell isolation

Primary MLECs were prepared as previously described^69^. Briefly, mouse lungs were dissected, minced and digested in 1 mg ml^−1^ type I collagenase (Worthington Biochem. Corp., #LS004196) for 1 h. Endothelial cells were purified using CD31 Ab-coated Dyna-beads and cultured in DMEM supplemented with 10% fetal bovine serum (FBS), 100 units/ml penicillin, 100 μg ml^−1^ streptomycin, 100 μg ml^−1^ endothelial cell mitogen (Biomedical Technologies, Inc, #BT-203), and 10 units per ml heparin. After 5–7 days, endothelial cells were further enriched by ICAM-2 Ab-coated Dyna-beads to more than 90% purity.

HUVECs were obtained from Yale Vascular Biology & Therapeutics (VBT) program and maintained in medium supplemented with 2% FBS, VEGF and other necessary growth factors and cytokines (EGM-2 BulletKit, Lonza Inc., Rockland, ME). The cells were verified by CD31 and VE-cadherin staining for endothelial cell identify. Wi-38 fibroblasts were purchased from ATCC and maintained in DMEM containing 10% FBS, 100 units per ml penicillin, 100 μg ml^−1^ streptomycin, 2 mM L-glutamine and 1 mM sodium pyruvate. All cells were free of mycoplasma contamination.

### Adenoviral constructs and recombinant protein

Full-length (FL) and cytoplasmic deleted (ΔCD) mouse Robo4 constructs were obtained by PCR from mouse Robo4 cDNA (Robo4FL: aa 1–1015; Robo4ΔCD: aa 1–522) and fused to mCherry (Supplementary Fig. 1). Rat Robo1 full length (Robo1FL) (aa 27–1,651) and Robo1ΔCD (aa 27–941) constructs were generated by PCR from rat cDNA and fused to IgK signal peptide (METDTLLLWVLLLWVPGSTGD) and GFP (Supplementary Fig. 1). Unc5BFL and truncated Unc5B constructs (ΔCD: aa 1–424; ΔDD: aa 1–852, ΔUPA: aa 1–652 and UPA: aa 1–538+688–838) were obtained by PCR from rat Unc5B cDNA and fused to GFP (Fig. 7e). The fusions were subcloned into pENTR1A vector (Invitrogen) and then transferred into pAd/CMV/V5/DEST using the Gateway Cloning System (Invitrogen, #V49320). To produce adenoviruses, the constructs were transfected into HEK293 cells and the adenovirus containing supernatants were collected. After titration, the viruses were used to infect cells.

Recombinant mouse sRobo4 (aa 1–478) construct was generated by PCR from mouse Robo4 cDNA and cloned into the eukaryotic expression vector pRK5 as fusion to a C-terminal 6x Histidine tag (sRobo4-His). The construct was transiently transfected into CHO cells to produce the protein. The recombinant protein was purified to >95% purity by affinity chromatography using NiNTA affinity purification (NiNTA Superflow, Qiagen) and reconstituted in PBS/0.2 M NaCl.

### ELISA

ELISA was performed using human anti Robo4–1 mAb^18^ and a biotinylated anti-Robo4 Ab (R&D Systems, #BAF2366) as coating and detection Ab, respectively. In brief, 96-well plates (Nunc) were precoated with 1 μg ml^−1^ anti-Robo4-1 mAb overnight, washed with PBST (0.05% Tween-20 in PBS) and blocked with PBS/2% goat serum/1% BSA. Thereafter, each well was incubated with 100 μl mouse serum sample for 2 h, 100 μl of 0.5 μg ml^−1^ biotinylated anti-Robo4 Ab (diluted in PBS/1% BSA) for 2 h and streptavidin-HRP (diluted in PBS/1% BSA) for 30 min at room temperature, with appropriate PBST washes after each incubation. The wells were developed with ELISA development substrate mix (R&D Systems) for 10–20 min, and the reaction was stopped with stop solution followed by absorbance reading at 450 nm. Concentrations of sRobo4 in mouse serum were calculated using a standard curve generated from mouse sRobo4-HIS.

### Immunoprecipitation

Cell culture supernatants, and human or mouse serum were precleared twice with protein A/G magnetic beads (Thermo Scientific, #88803) and incubated with anti-Robo4-1 mAb in the presence of appropriate protease inhibitors overnight at 4 °C, followed by 1 h incubation with protein A/G beads. Precipitates were washed three times in PBS, resolved in 2XLaemmli's sample buffer and sRobo4 was detected by Western blot using R&D anti-Robo4 Ab.

### Whole-mount staining of retinas

The eyes of mouse pups were fixed in 4% PFA for 20 min. The retinas were dissected and incubated with fluorescent labelled-IsoB4 in Pblec buffer (1 mM MgCl_2_, 1 mM CaCl_2_, 0.1 mM MnCl_2_, 1% Triton X-100 in PBS) overnight. After 3 × 15 min washing with PBS, the retinas were mounted in fluorescent mounting medium (DAKO Inc.).

### Oxygen-induced retinopathy

OIR was performed as described^20^,^21^. Briefly, the breeding mother and P7 neonatal pups of both genders were exposed to 75% O_2_ until P12. The pups were then exposed to room air for an additional 5 days until P17. In some experiments, P12 OIR pups were subjected to intraperitoneal (I.P.) injection of 300 μg g^−1^ recombinant mouse sRobo4 from P12 to P16. Eyes were collected at P17 and the retinas were stained with IsoB4. The avascular, sprouting, tuft and total retina areas were measured using ImageJ.

To test OIR retinal vessel leakage, a mixture of 70 kDa fixable rhodamine-dextran (200 mg kg^−1^ body weight) and fluorescent Alexa 647-conjugated IsoB4 (5 mg kg^−1^ body weight) was injected into P17 OIR mice (5–6 g) retro-orbitally. Mice were sacrificed after 5 min or as indicated. The eyes were fixed in 4% PFA for 20 min. The retinas were dissected and incubated with fluorescent Alexa 488-conjugated IsoB4 in Pblec buffer for 10 h. Stained retinas were flat-mounted and imaged with Alexa 488, rhodamine and Alexa 647 channels. To quantify dextran retained in vessels, mean fluorescent intensity of rhodamine in Alexa 647-labelled vessel area was measured using image J, followed by normalization with Alexa 647 intensity.

### Miles vascular permeability assay

Six-to-eight week old mice of both genders were injected intravenously with 100 μl PBS containing 1% Evans blue. 15 min afterwards, 50 ng VEGF (in 20 μl saline) or 50 ng histamine (in 20 μl PBS) was injected intradermally into the shaved back skin. After 30 min, the animals were perfused with 20 ml PBS, and back skin was dissected and imaged. To extract the dye, skin biopsies were collected, dried, weighed and incubated in formamide solution at 56 °C overnight. The absorbance of the extracts was measured with a spectrophotometer at 620 nm. Evans blue content was calculated using a standard curve of Evans blue in formamide and normalized to saline/PBS injected control wild-type mice.

### *In vivo* wound healing assay

Mouse *in vivo* wound healing assay was described previously^70^. Eight-to-ten week old male mice were shaved and depilated on the back. Wounds were created by a 6-mm biopsy punch on the back skin. Wound images were acquired with a Leica M125 microscope equipped with a digital camera on days 0 and 7. Wound areas were measured by ImageJ and presented as percentage of the initial wound area at day 0.

### *In vitro* sprouting assay

*In vitro* endothelial cell sprouting assays were performed as described^70^. Briefly, a layer of fibrin was made on the bottom of 24-well plates by mixing solubilized fibrinogen (10 mg ml^−1^ in EBM-2) with thrombin (final concentration 1 U). SiRNA-transfected and adenovirus infected HUVECs were suspended in fibrinogen solution and plated on top of the fibrin gel. After the upper layer of gel was solidified, Wi-38 fibroblasts were plated on the top. Cells were replenished with EBM-2 supplemented with 2% FBS in the presence of VEGF or Slit2 for 5 days. After trypsinizing fibroblasts on the top, sprouts were labeled with 4 mg ml^−1^ Calcein (Life Technologies) for 1 h, photographed with a fluorescence microscope and the area covered by endothelial sprouts measured by ImageJ.

### *In vitro* endothelial permeability assay

HUVEC permeability assay was performed in 24-well plates containing transwell inserts (6.5-mm diameter, 0.4-μm pore size polycarbonate filters, Corning Costar Corporation) according to manufacturer's instruction. In brief, HUVECs were transfected with siRNA on day 0 and infected with GFP or Robo4 adenovirus on day 1. On day 2, cells were plated onto fibronectin (10 μg ml^−1^)-coated transwell inserts at a density of 150,000 cells per cm^2^ and cultured in complete media for 3 days to allow formation of cell monolayer. To test transwell permeability, FITC-dextran (70 kDa, Molecular Probes) with final concentration of 1 mg ml^−1^ and 5 nM VEGF was added into the upper compartment of the inserts. After 30 min, FITC-dextran intensity in the medium of the lower compartment was measured using a fluorescence spectrophotometer.

### *In vitro* wound healing assay

HUVECs were transfected and infected with the indicated siRNA and adenovirus. After 48 h, cells were plated into 6-well plates, cultured until confluence and starved overnight in 0.2% FBS/EBM-2. Scratch wounds were created with a 200-μl pipette tip. The wounded cell monolayer was cultured in 0.2% FBS/EBM-2 supplemented with 6 nM VEGF-A. Cell-free areas were photographed at 0 h and 20 h post wounding under an inverted light microscope connected with a digital camera.

### Quantitative real-time PCR

Total RNAs were purified from HUVECs using the RNeasy Plus Mini Kit (Qiagen, #74134) and reverse transcribed to cDNAs using IScript Reverse Transcriptase III (Bio-Rad, #170–8891) according to manufacturers' instructions. QRT-PCR was performed using the resulting cDNAs and the corresponding primers (Supplementary Table 2). The data were first normalized to *GAPDH,* and the relative expression levels of different genes were calculated.

### Signalling studies

HUVECs were transfected with 20 pmol siRNA per well in 6-well plates using RNAiMax transfection reagent (Invitrogen, #13778) according to the manufacturer's instruction. Infection with GFP, Robo4 or Unc5B adenovirus was done one day after siRNA transfection. After 48 h, cells were starved 10 h to overnight in EBM-2 supplemented with 0.2% FBS and treated with 1 μg ml^−1^ (6 nM) recombinant Slit2 or 25 ng ml^−1^ (3 nM) VEGF-A. For MLEC signalling experiments, primary MLECs were starved 10 h to overnight in DMEM containing 2% FBS and stimulated with 5 nM VEGF-A. Cell lysates were collected at the indicated time points and subjected to 4–15% SDS–PAGE followed by immunoblotting using appropriate primary and second Abs. See Supplementary Fig. 8 for all uncropped immunoblots.

### Statistics

No statistical methods were used to determine sample size before experiments. Animals were selected for animal experiments based on their genotypes and proper age and gender as described in ‘Methods' section, which was a pre-established criterion before the experiment. No randomization and blinding were used. Samples with equal variances were tested using Mann–Whitney *U* test or two-tailed Student's *t*-test between groups. When the variances were unequal, samples were tested using Welch's *t*-test. *P* value <0.05 was considered to be statistically significant.

### Data availability

All data that support the findings of this study are available within the article and its Supplementary Information File and from the corresponding author upon reasonable request.

## Additional information

**How to cite this article:** Zhang, F. *et al*. The Robo4 cytoplasmic domain is dispensable for vascular permeability and neovascularization. *Nat. Commun.* **7,** 13517 doi: 10.1038/ncomms13517 (2016).

**Publisher's note**: Springer Nature remains neutral with regard to jurisdictional claims in published maps and institutional affiliations.

## Supplementary Material

Supplementary Information Supplementary Figures 1-8 and Supplementary Tables 1-3.
