# Supplementary material for: The Robo4 cytoplasmic domain is dispensable for vascular permeability and neovascularization
Source: Nat Commun. 2016 Nov 24;7:13517. doi: 10.1038/ncomms13517 (PMC5123080; doi:10.1038/ncomms13517)
Supplement: Supplementary Information — Supplementary Figures 1-8 and Supplementary Tables 1-3. [file ncomms13517-s1.pdf]

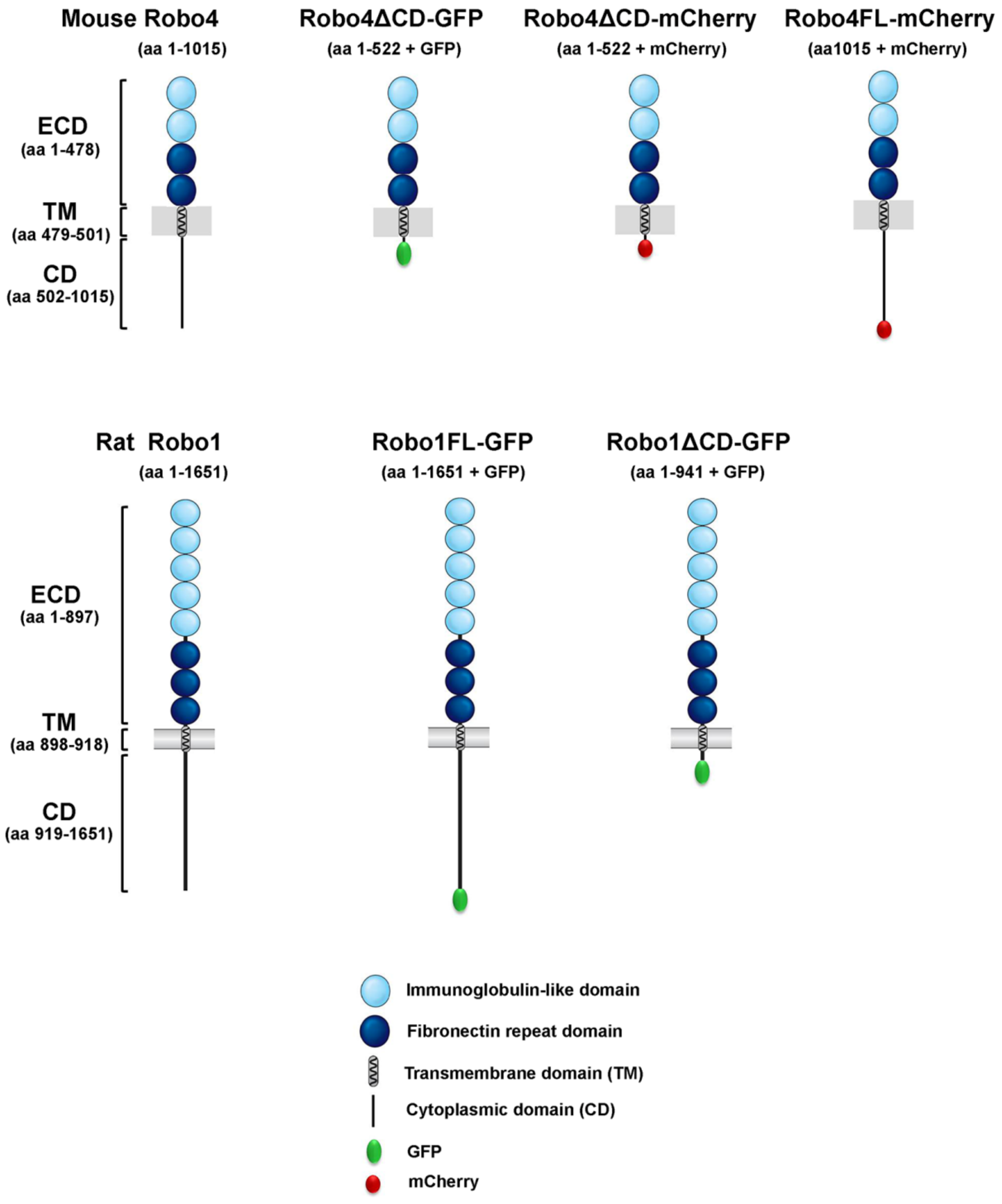

Supplementary Fig. 1. Schematic diagram showing Robo constructs used in the study.

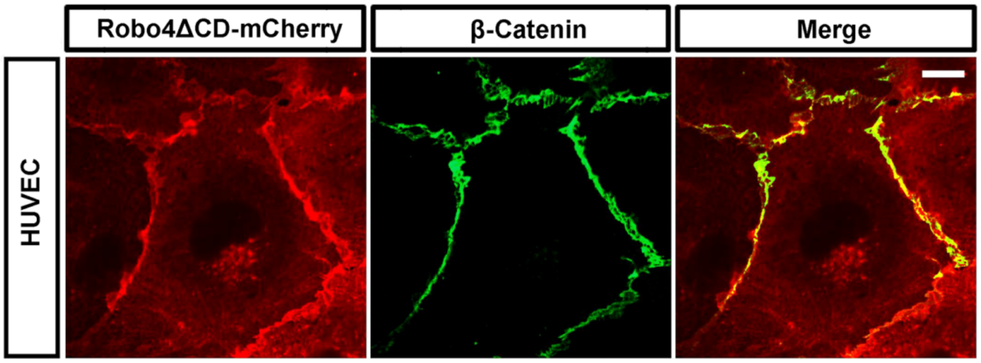

Supplementary Fig. 2. Membrane and junctional labelling of HUVECs infected with Robo4ΔCD-mCherry adenovirus counterstained with anti-β-catenin antibody. Scale bar: 10 μm.

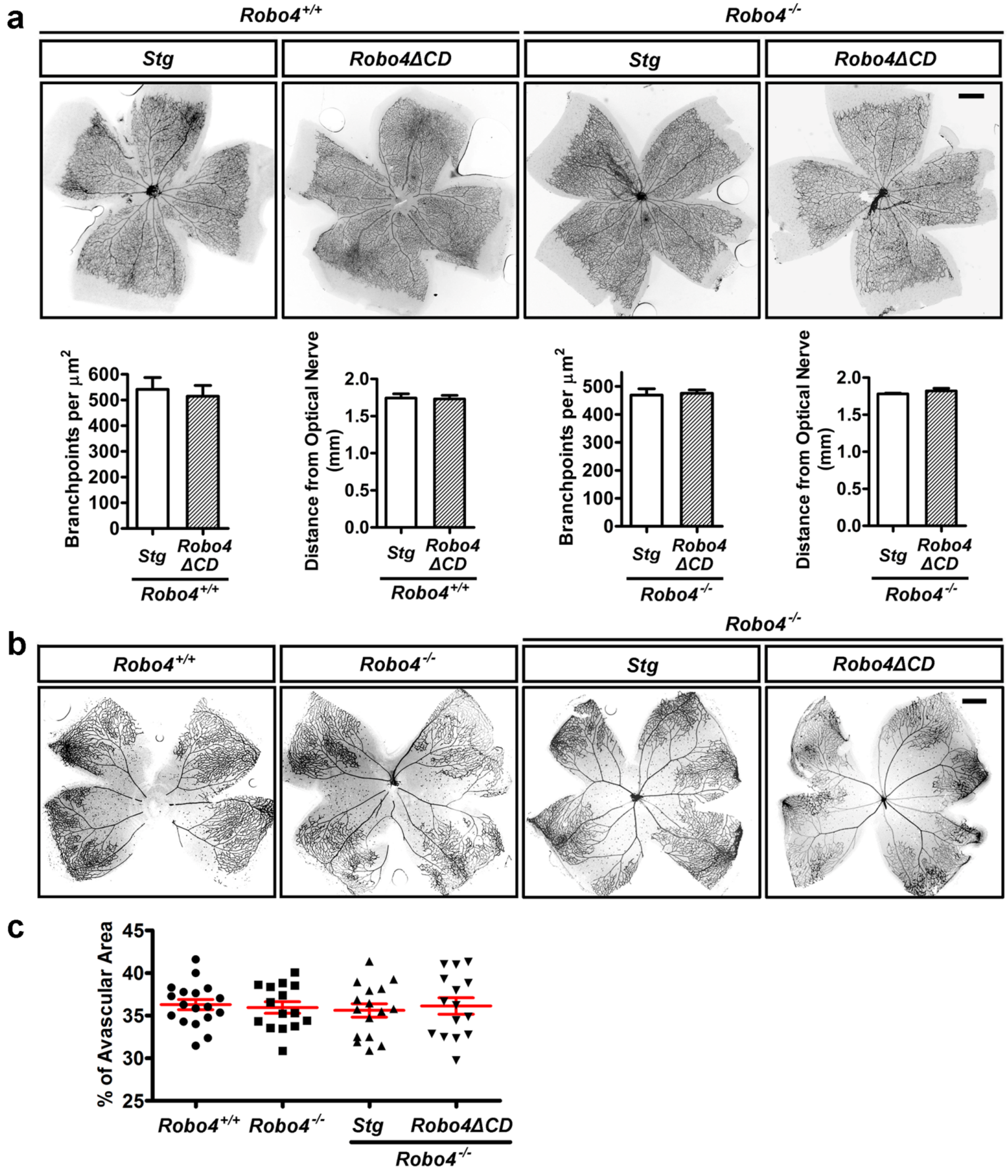

Supplementary Fig. 3. Robo4ΔCD does not affect developmental retinal angiogenesis and hyperoxia-induced vaso-obliteration.

(a) Developmental retinal angiogenesis analysis. Upper panel: IsoB4-stained P7 retinal flat mounts of mice with the indicated genotypes. Lower panel: quantifications of number of branchpoints and vascular progression of the retina vasculature. N= 4-10 mice in each group. Error bars: S.E.M. No significant differences were observed using Mann-Whitney U test.

(b) Robo4 or Robo4ΔCD does not affect vaso-obliteration in retina after OIR. IsoB4 stained P12 retinal flat mounts of mice with the indicated genotypes after 5 days of 75% hyperoxia exposure.

(c) Quantifications of avascular area in the retinas shown in **(b)**. Each dot represents a retina. N= 15-18 retinas (8-9 mice) per group. Error bars: S.E.M. No significant differences were observed using Mann-Whitney U test.

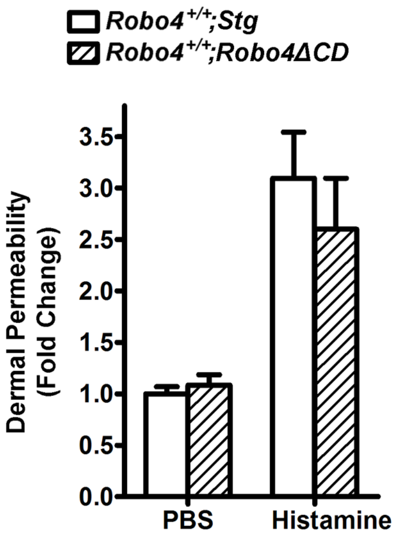

Supplementary Fig. 4. Dermal permeability analysis.

Quantifications of Evans blue dye in the skin 30 min after histamine stimulation. N= 8-11 mice per group. Error bars: S.E.M. Histamine induced dermal permeability was not significantly different between *Robo4$^{+/+}$;Stg* and *Robo4$^{+/+}$;Robo4 Δ CD* mice, as determined using Mann-Whitney U test.

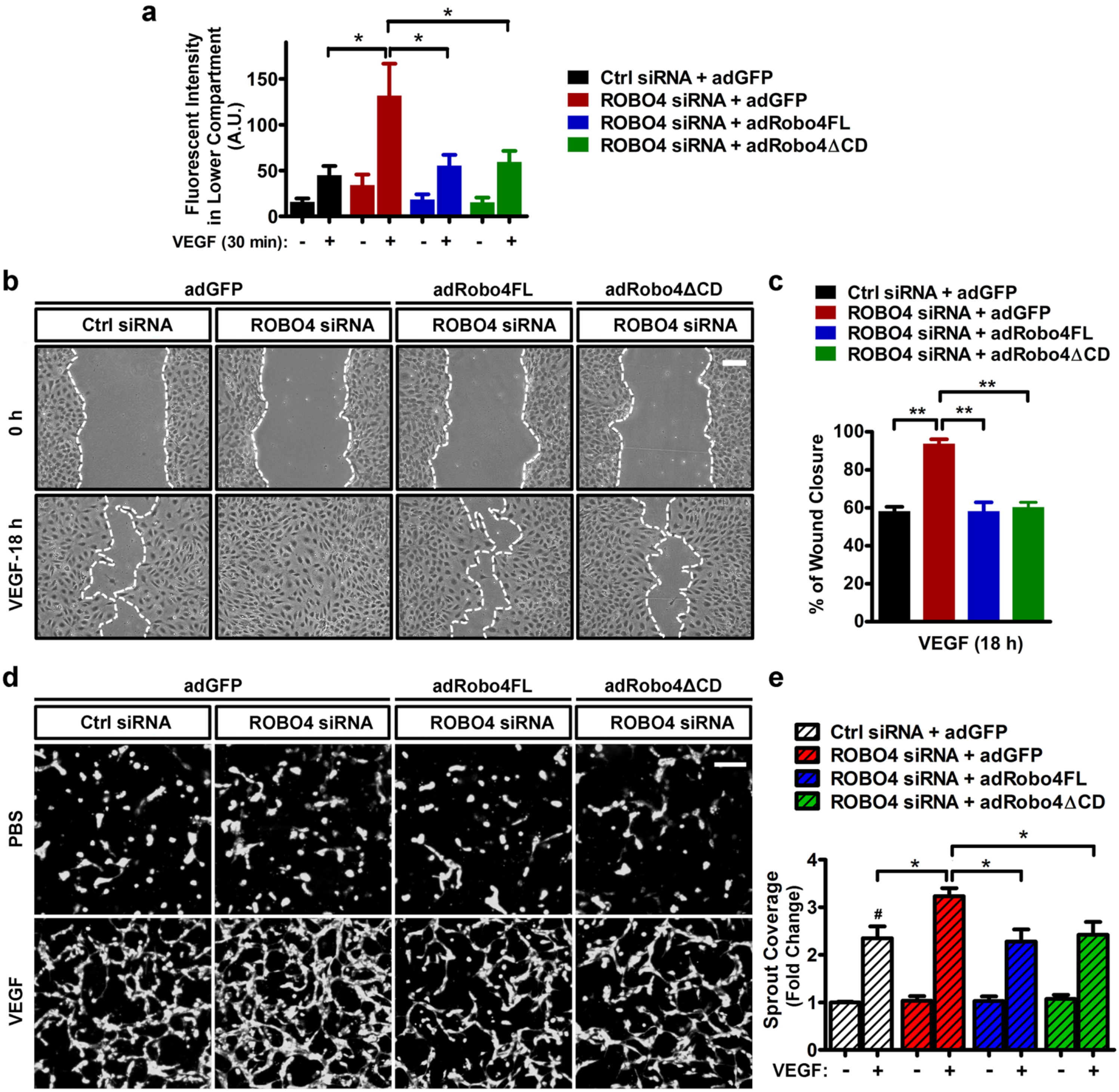

Supplementary Fig. 5. Robo4ΔCD inhibits VEGF-induced HUVEC monolayer permeability, wound closure and vascularisation in 3D fibrin gels.

(a) HUVEC transwell permeability assay. HUVECs were treated with siRNAs and virus as indicated and cultured for 3 days in transwell inserts. After stimulation with 5 nM VEGF for 30 min, FITC-dextran leakage into the lower compartment of the inserts was assessed. VEGF induced FITC leakage was significantly enhanced by *ROBO4* siRNA. Expression of siRNA resistant Robo4FL and Robo4ΔCD in *ROBO4* siRNA transfected cells rescued the enhanced FITC leakage. N=4 experiments. The data represent mean ± standard deviation. * $p < 0.05$, Mann-Whitney U test.

(b-c) Representative images and quantifications of HUVEC scratch wound healing assay. Cells were treated with siRNAs and virus as indicated and then stimulated with 6 nM VEGF. Images were taken at 0 h and 18 h after scratching. VEGF induced migration was significantly enhanced by *ROBO4* siRNA and decreased in Robo4FL and Robo4ΔCD transfected cells. N=6 scratches in 3 independent experiments. Error bars: S.E.M. **, $p < 0.01$, Mann-Whitney U test.

(d-e) HUVEC sprouting in 3D fibrin gels and the corresponding quantification. Cells were treated with siRNAs and virus as indicated and then stimulated with 6 nM VEGF for 120 h. #: VEGF significantly induces HUVEC sprouting in 3D fibrin gels. VEGF induced sprouting was significantly enhanced by *ROBO4* siRNA and decreased in Robo4FL and Robo4ΔCD transfected cells. N= 4 experiments. Error bars: S.E.M. *, $p < 0.05$, Mann-Whitney U test.

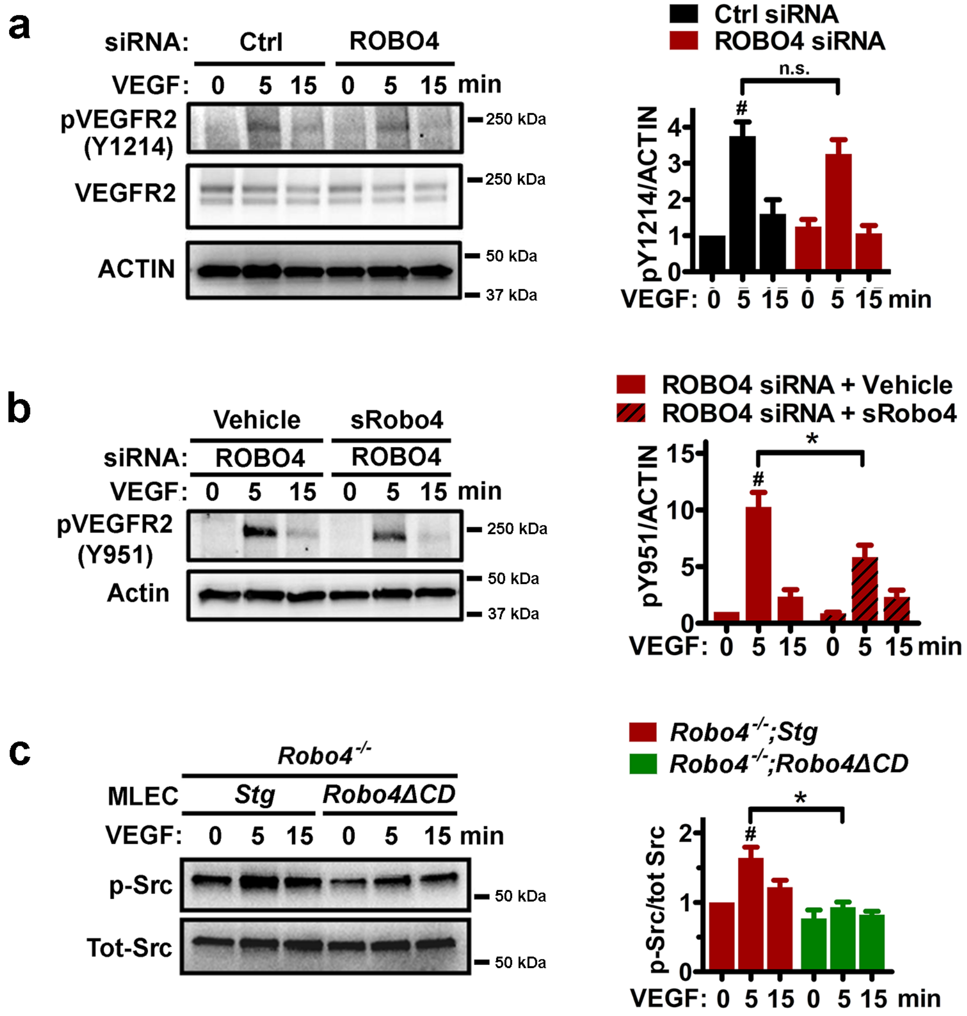

Supplementary Fig. 6. ROBO4 cytoplasmic domain is dispensable for VEGF signalling.

(a) Western-blot analysis (left) and quantifications (right, n=4) of phospho-VEGFR2 (Y1214) in HUVECs transfected with the indicated siRNAs and treated with 3 nM VEGF. #: VEGF significantly induces pY1214 at 5 min. Error bars: S.E.M. n.s., not significant, Student's *t*-test.

(b) Western blot analysis (left) and quantifications (right, n=5) of phospho-VEGFR2 (Y951) in HUVECs transfected with *ROBO4*siRNA, treated with 100 μg/ml recombinant mouse sRobo4 or vehicle (PBS/0.2 M NaCl) and stimulated with 3 nM VEGF. #: VEGF significantly induces pY951 at 5 min. sRobo4 inhibits excessive pY951 in *ROBO4*-silenced cells. Error bars: S.E.M. *, $p < 0.05$, Student's *t*-test.

(c) Western-blot analysis (left) and quantifications (right, n=5) of phospho-Src (p-Src) in MLECs of *Robo4$^{-/-}$;Stg* and *Robo4$^{-/-}$;Robo4ΔCD* mice after 6 nM VEGF stimulation. #: VEGF significantly induces p-Src in MLECs of *Robo4$^{-/-}$;Stg* mice at 5 min. Src activation is significantly decreased in *Robo4$^{-/-}$;Robo4ΔCD* mice. Error bars: S.E.M. *, $p < 0.05$, Student's *t*-test.

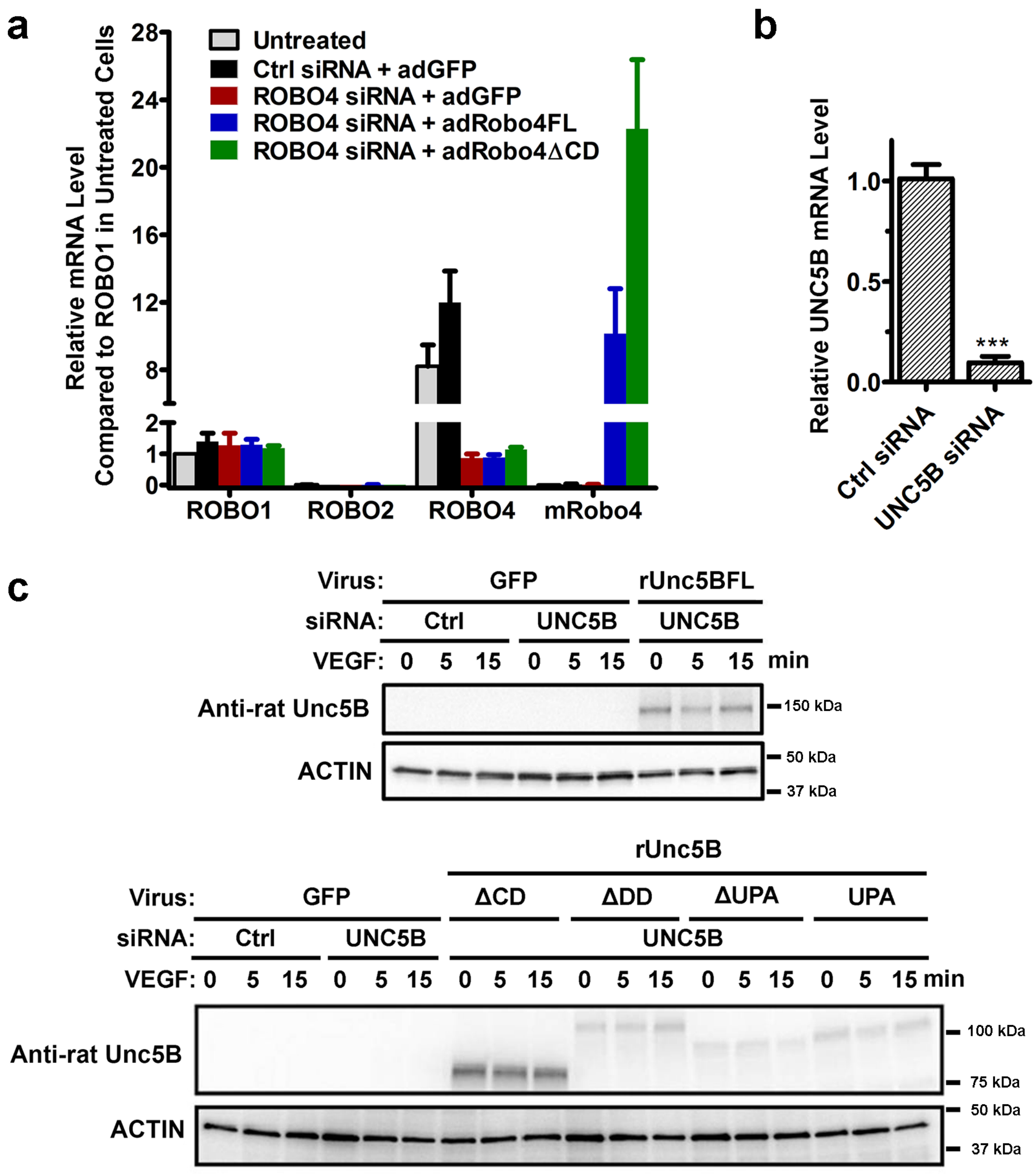

Supplementary Fig. 7. ROBO4 and UNC5B expression analysis.

(a) QPCR analysis of *ROBO* mRNA expression levels in HUVECs transfected with Ctrl or *ROBO4* siRNA and infected by adenovirus containing GFP (adGFP), adRobo4 Δ CD or adRobo4FL. *ROBO1* mRNA level in untreated HUVECs was set as 1. N=3 experiments.

(b) QPCR analysis of *UNC5B* mRNA expression levels in HUVECs transfected with Ctrl or *UNC5B*siRNA. Averaged *UNC5B* mRNA level in Ctrl siRNA transfected HUVECs was set as 1. N=3 independent experiments. Error bars: S.E.M. ***, $p < 0.001$, Student's *t*-test.

(c) Western blot analysis of siRNA-resistant rat Unc5BFL (upper panel) and truncated Unc5B (lower panel) in HUVECs with indicated siRNA transfection, adenovirus infection and 3 nM VEGF stimulation. Note that the blots are corresponding to those shown in Fig.7e and 7g.

Supplementary Fig. 8. Uncropped Immunoblots.

Uncropped Immunoblots for Fig.1c

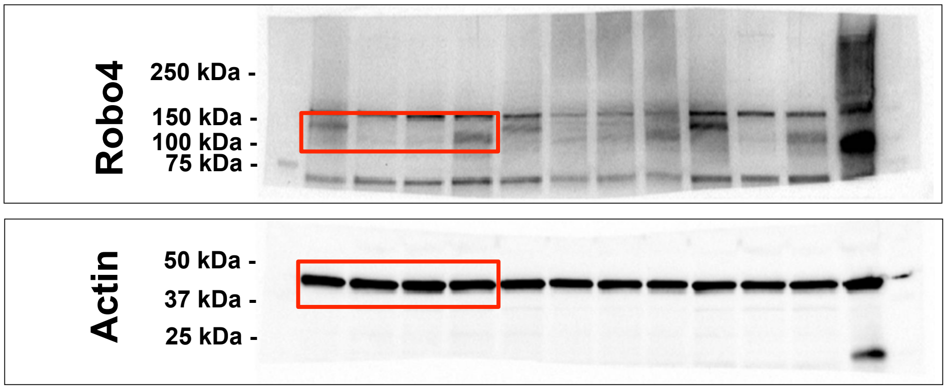

Uncropped Immunoblots for Fig.1e

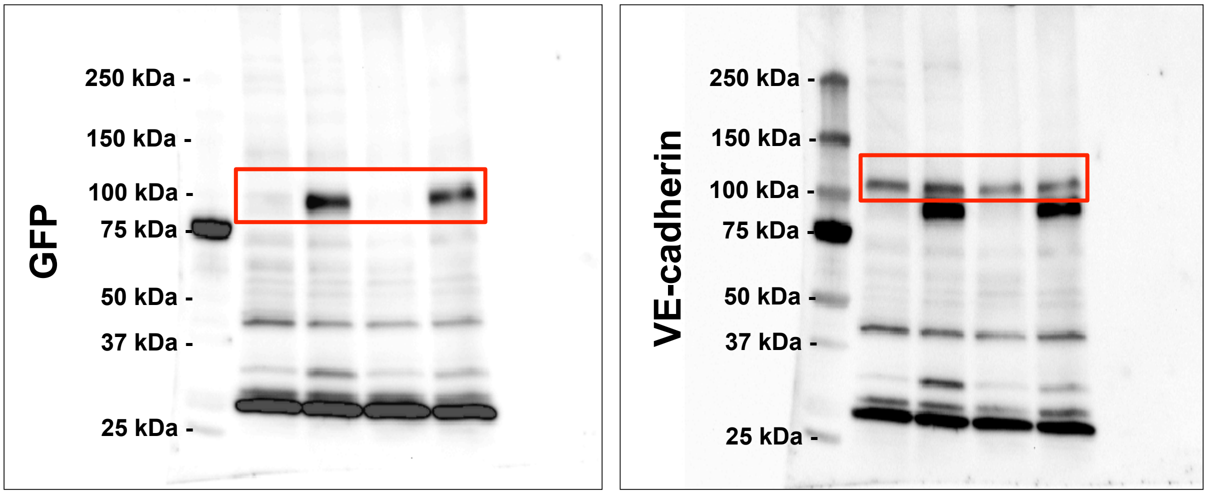

Uncropped Immunoblots for Fig.1j

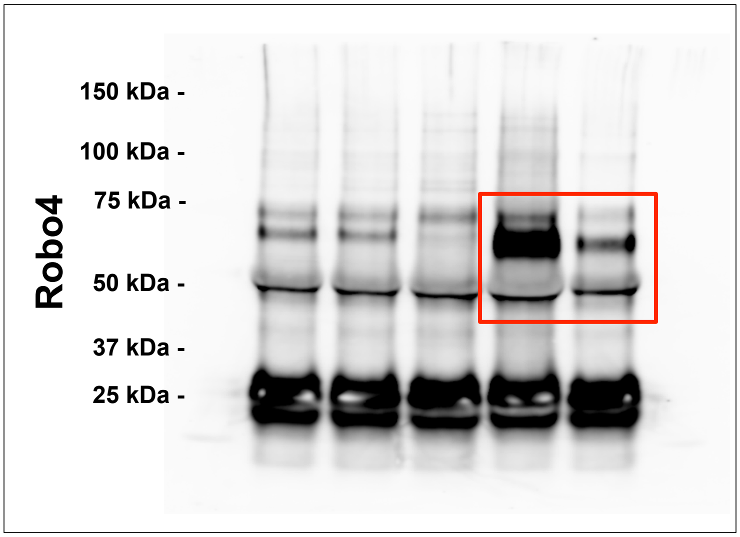

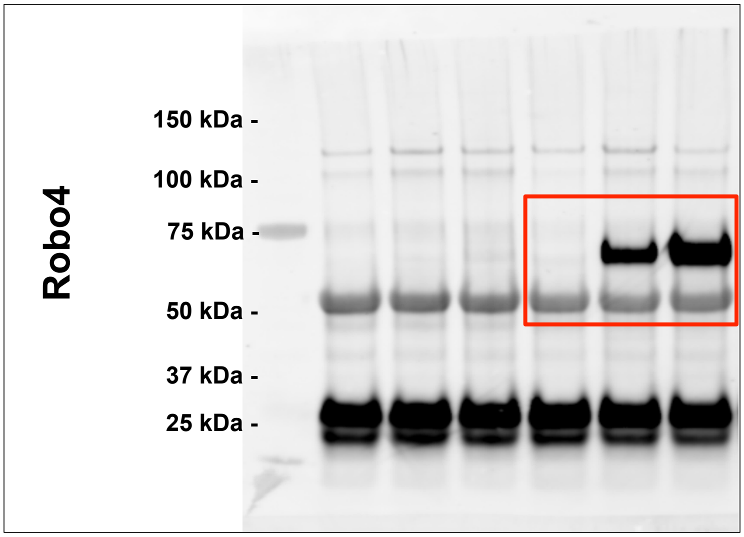

Uncropped Immunoblots for Fig.1k

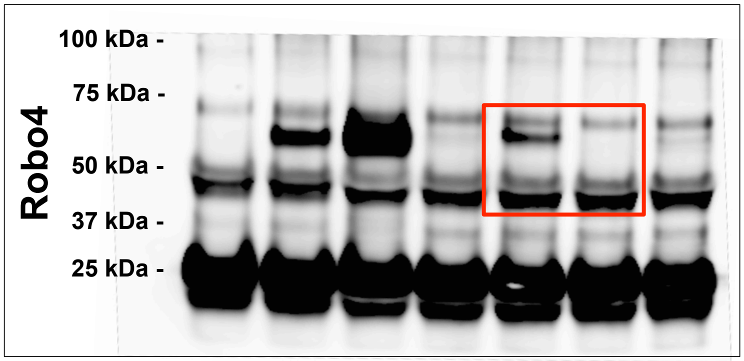

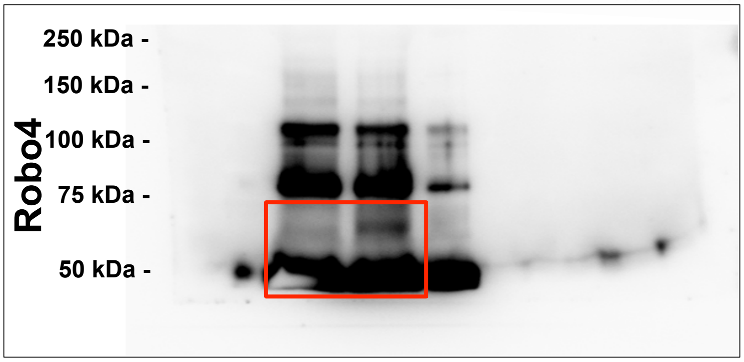

Uncropped Immunoblots for Fig.5a

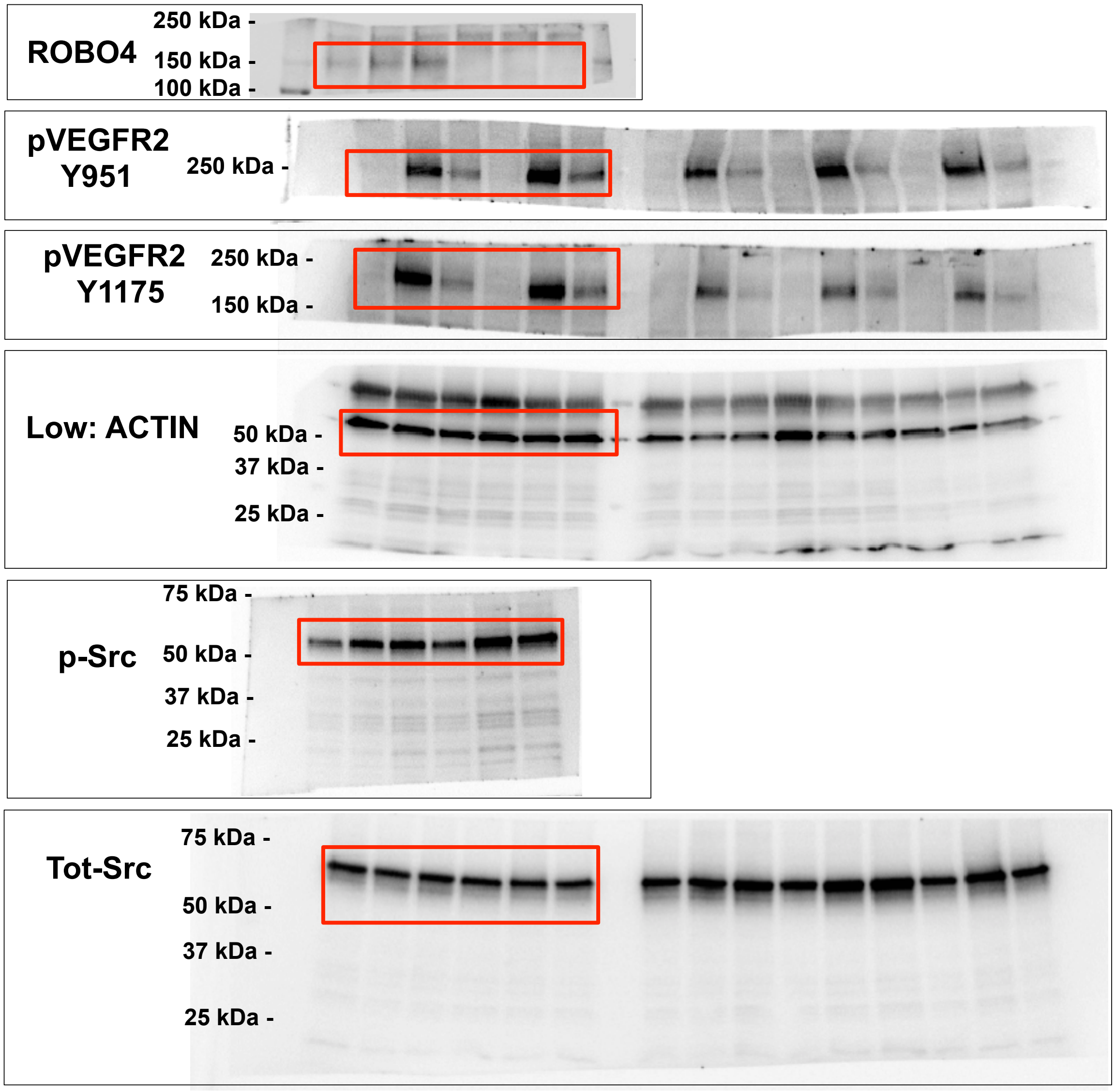

Uncropped Immunoblots for Fig.5e

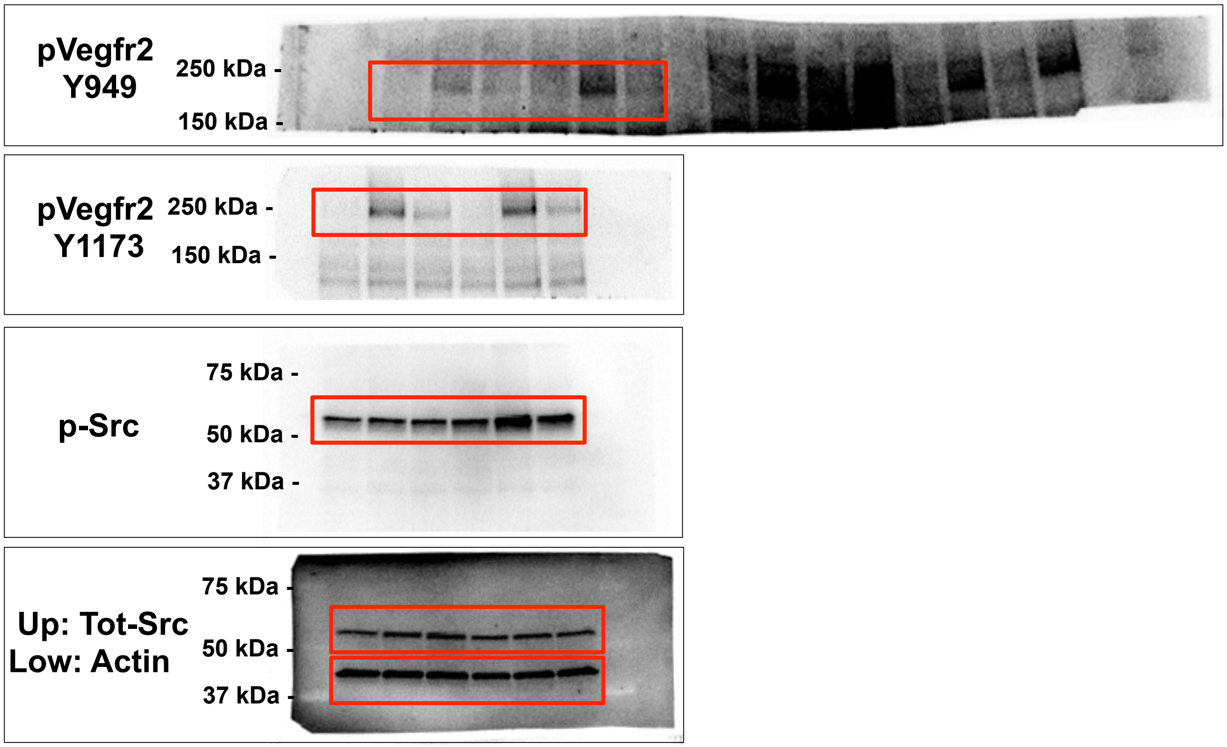

Uncropped Immunoblots for Fig.5i

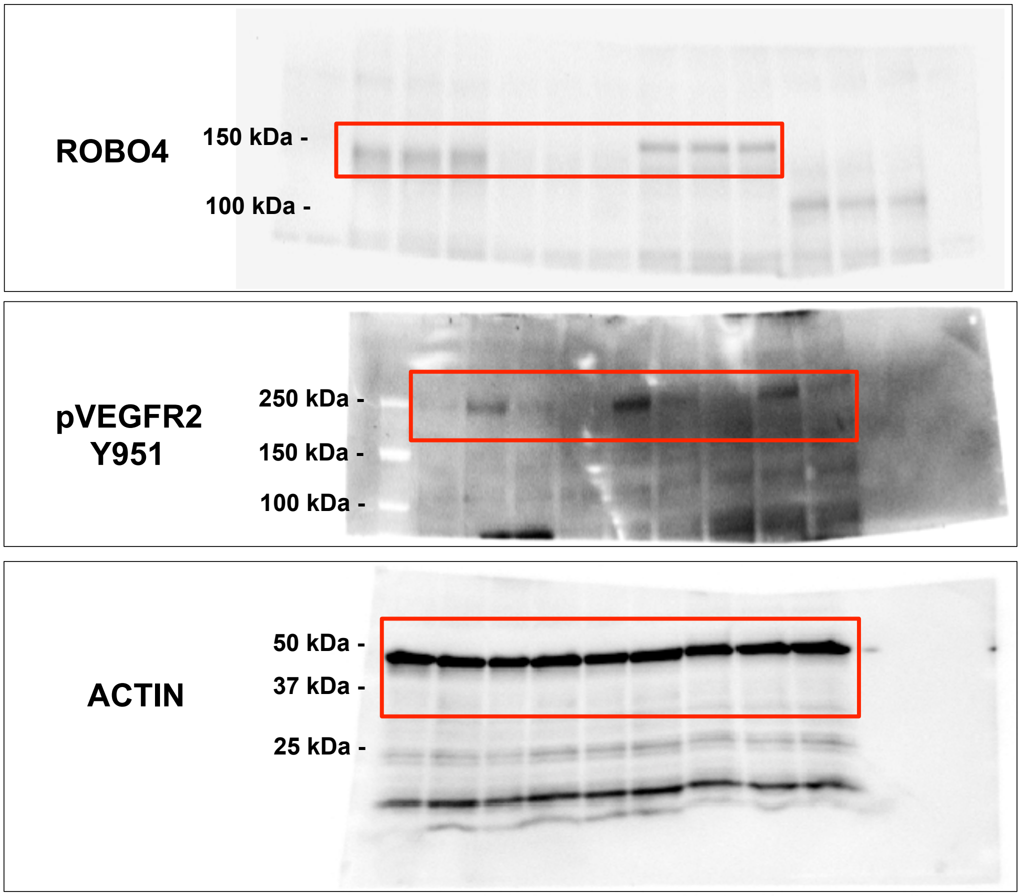

Uncropped Immunoblots for Fig.5k

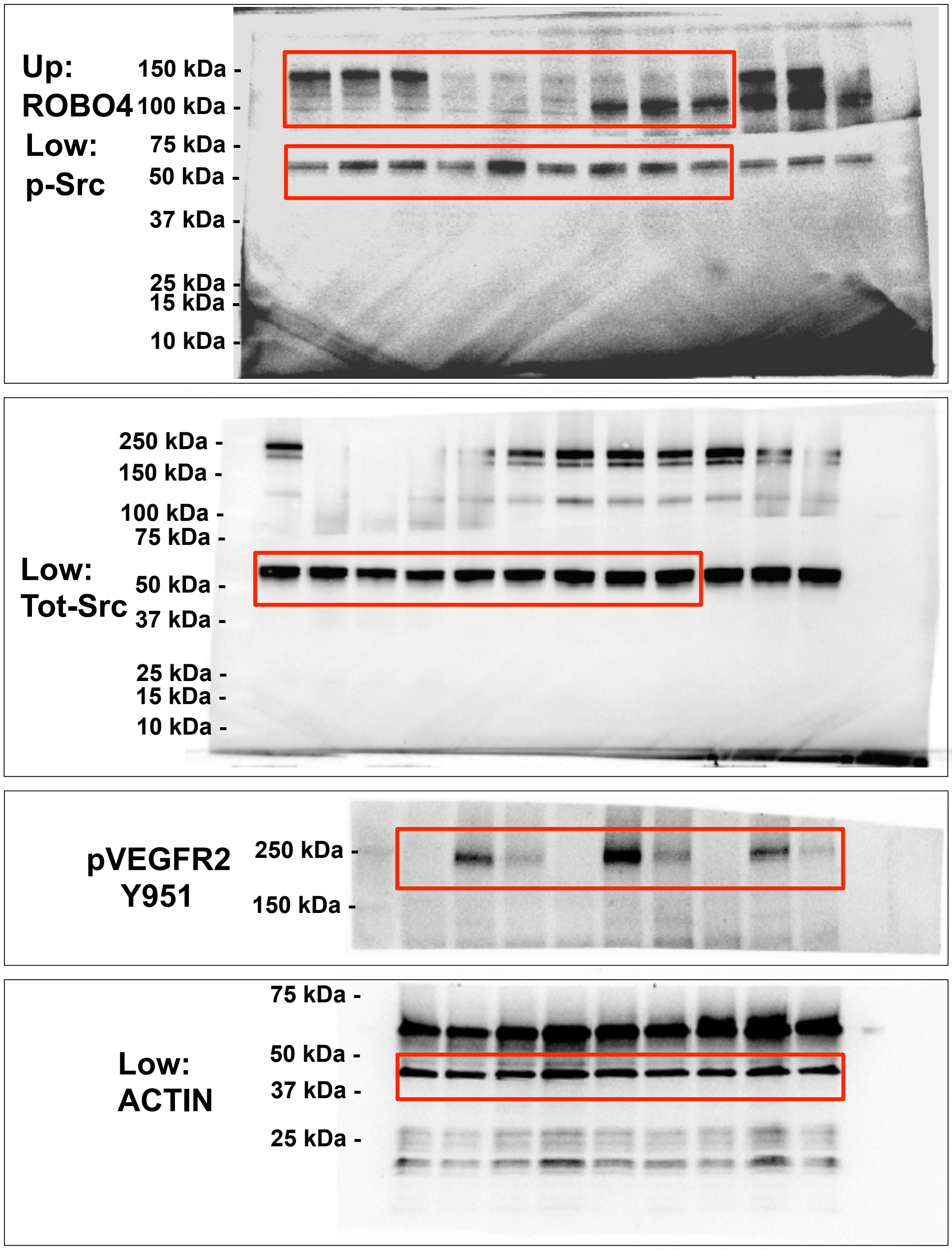

Uncropped Immunoblots for Fig.5m

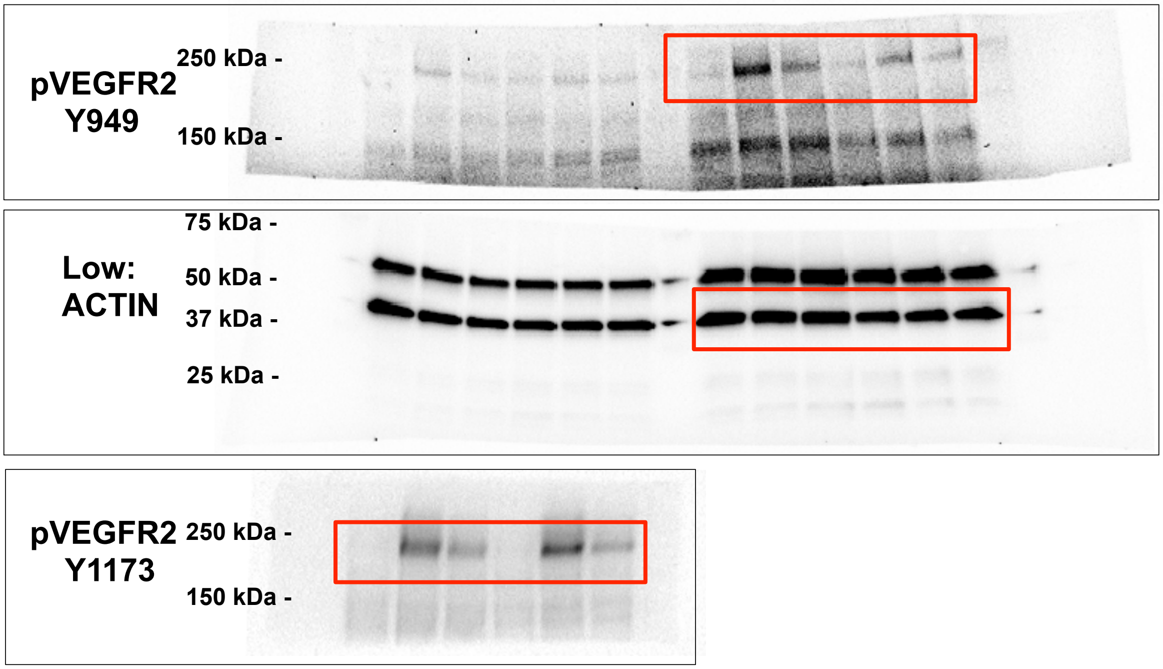

Uncropped Immunoblots for Fig.6b

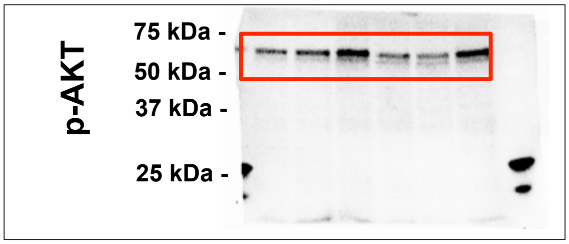

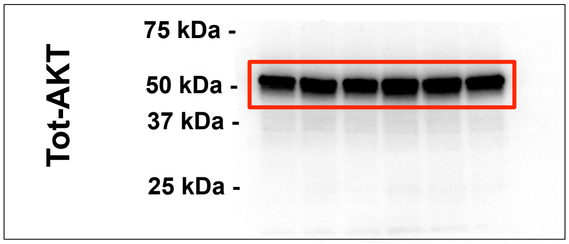

Uncropped Immunoblots for Fig.6c

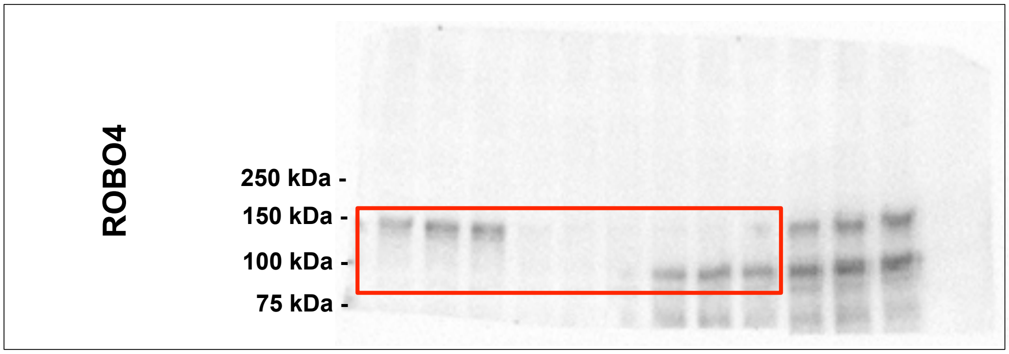

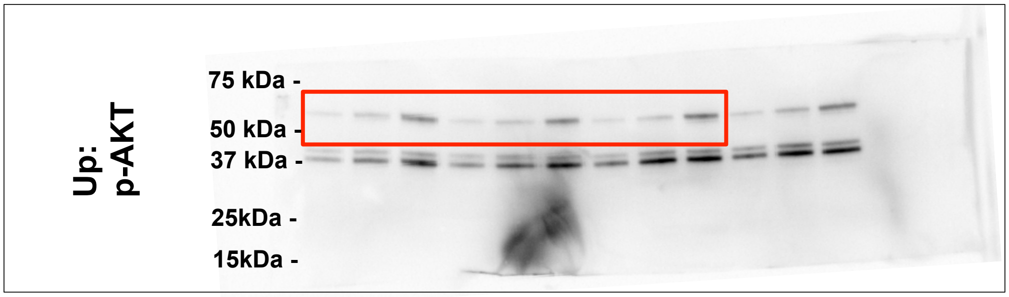

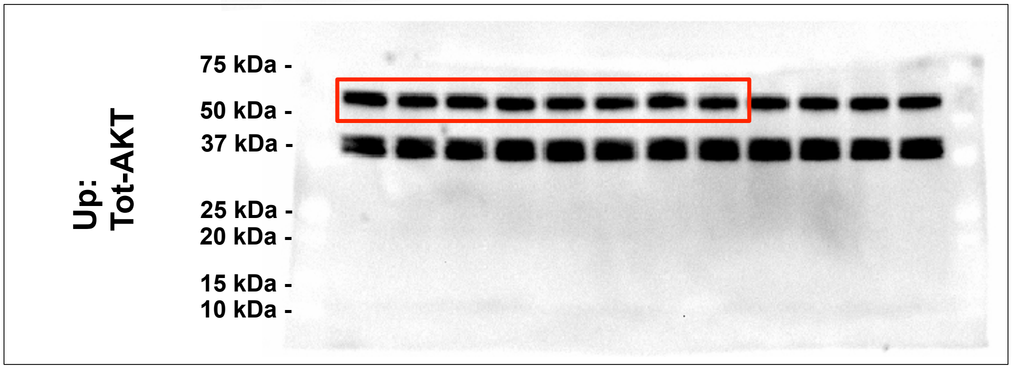

Uncropped Immunoblots for Fig.6e

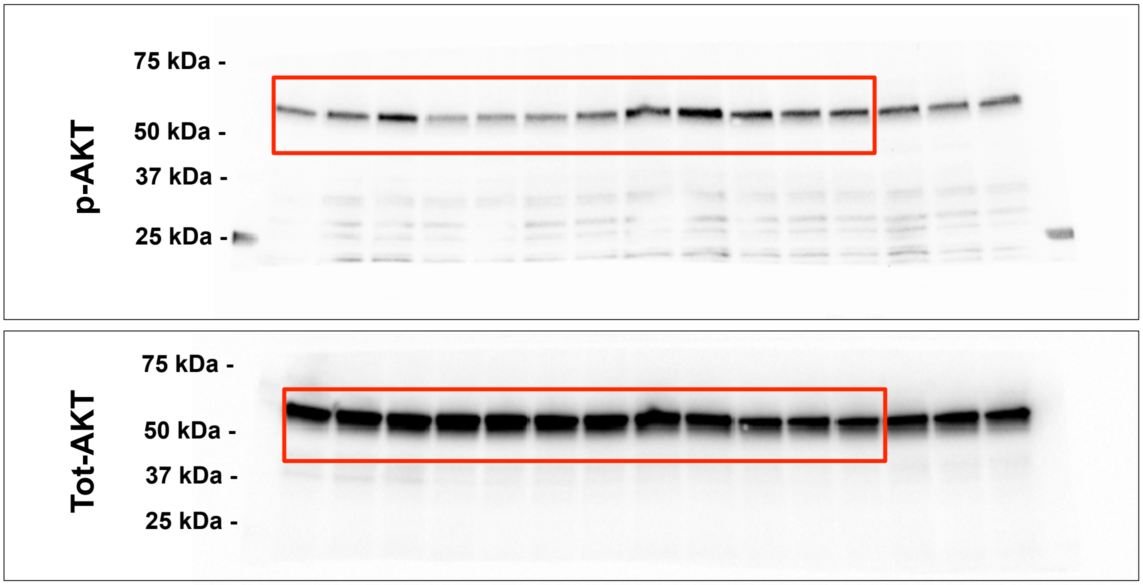

Uncropped Immunoblots for Fig.7b

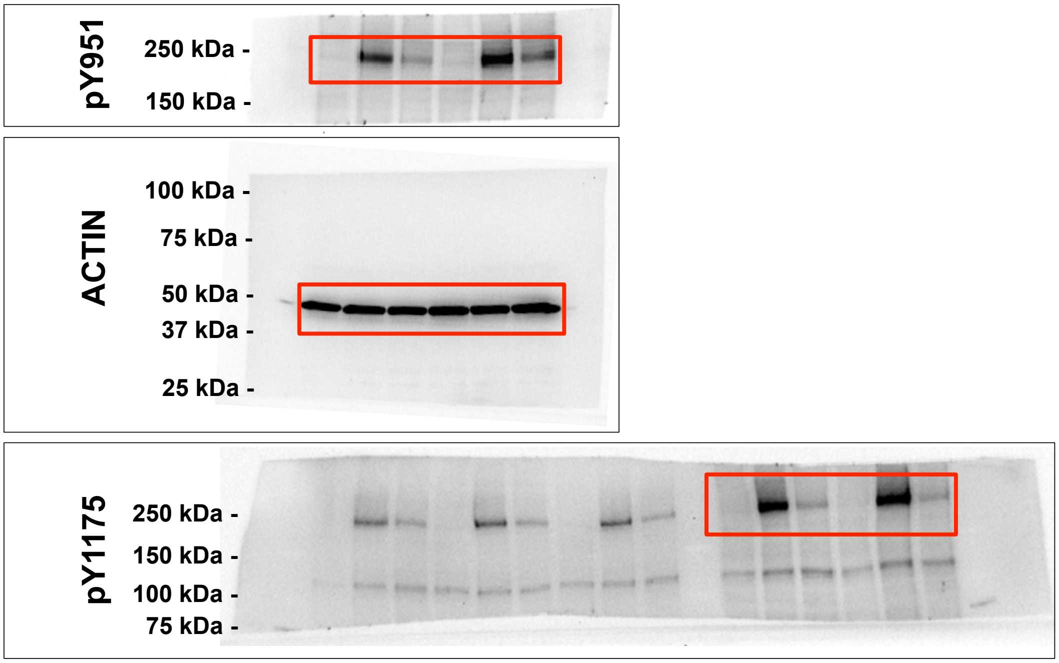

Uncropped Immunoblots for Fig.7e

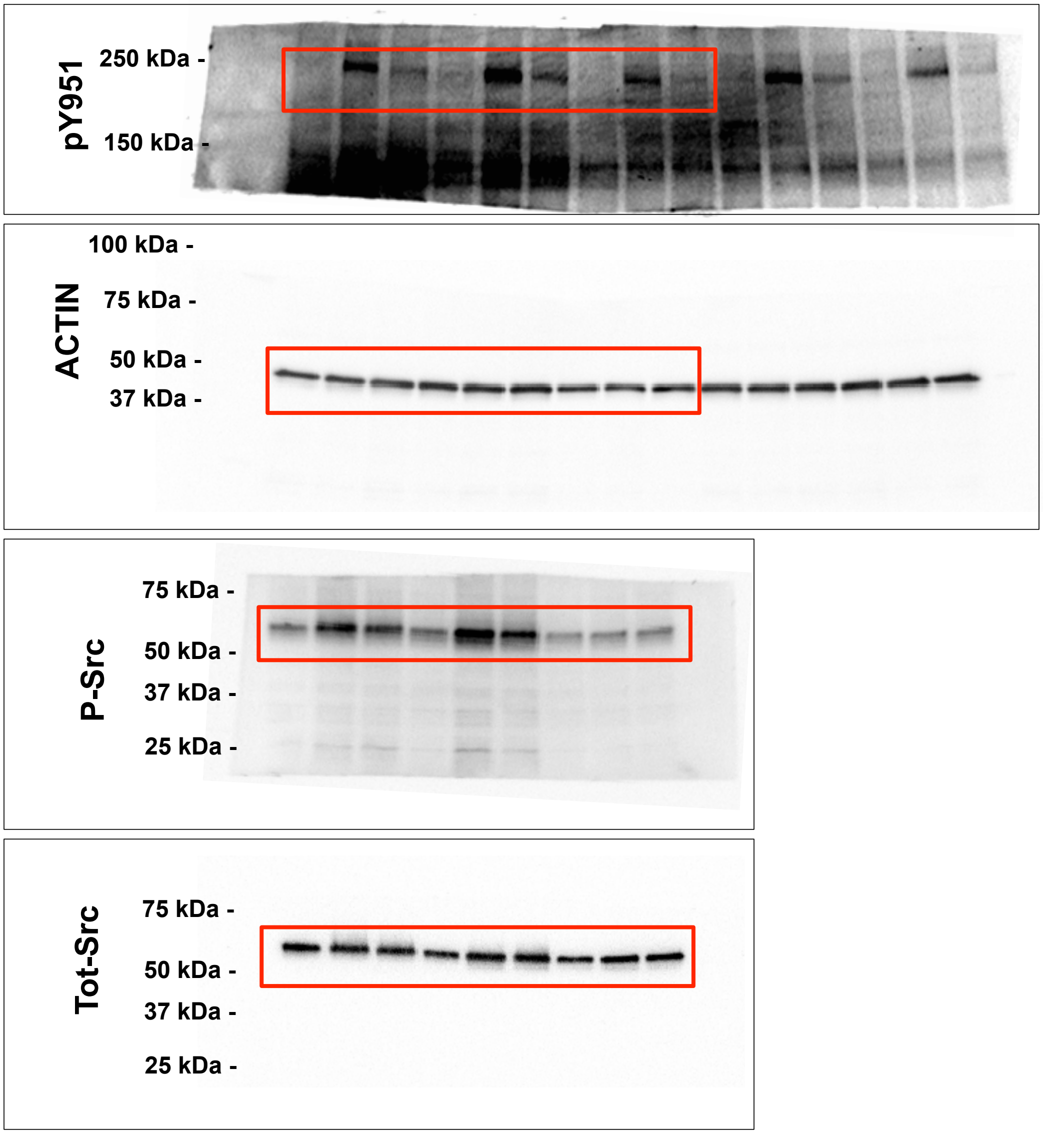

Uncropped Immunoblots for Fig.7g

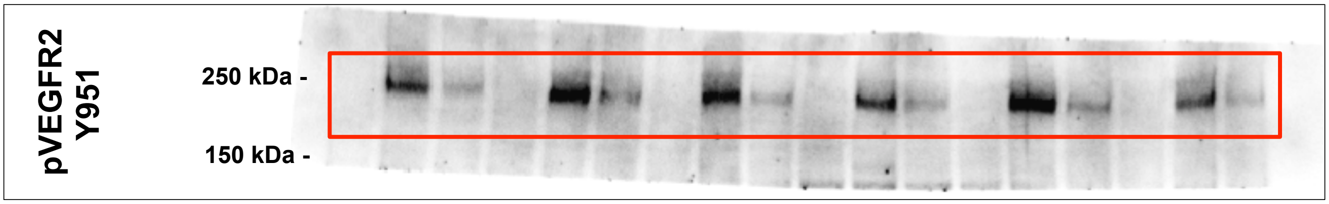

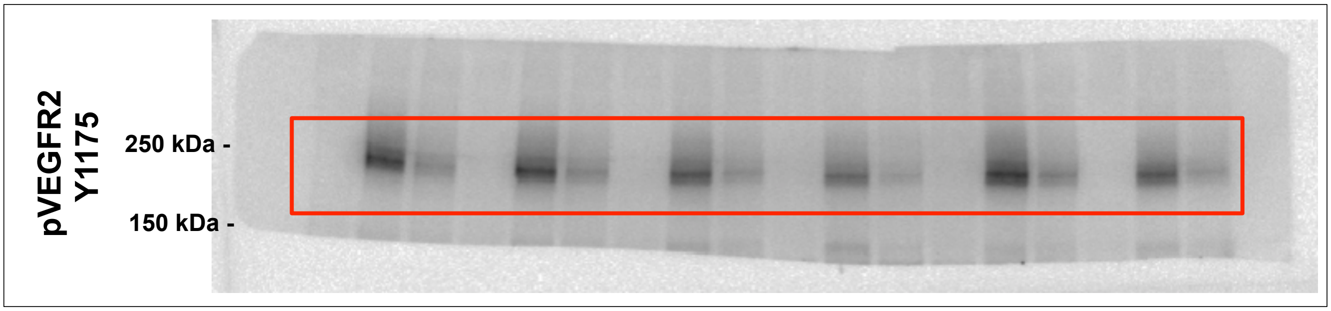

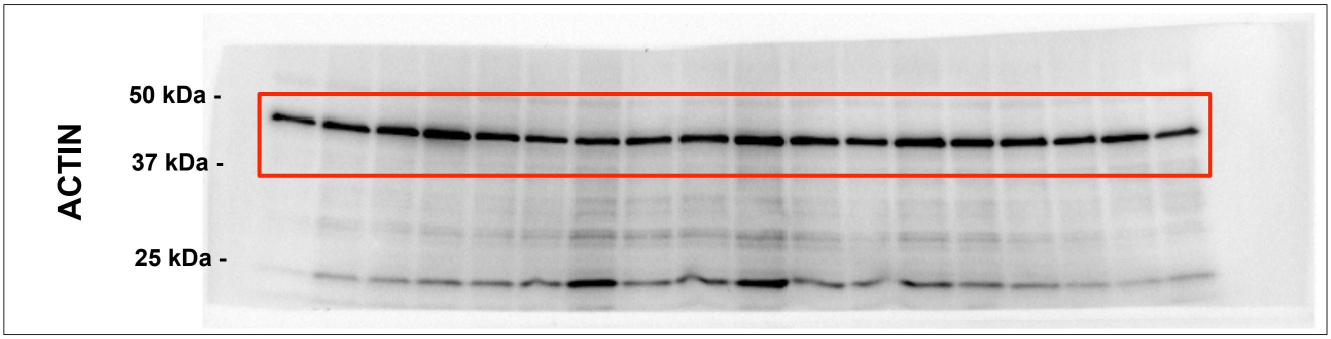

Uncropped Immunoblots for Fig.7i

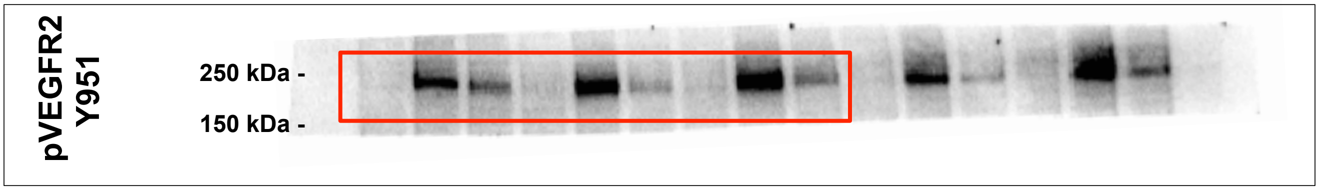

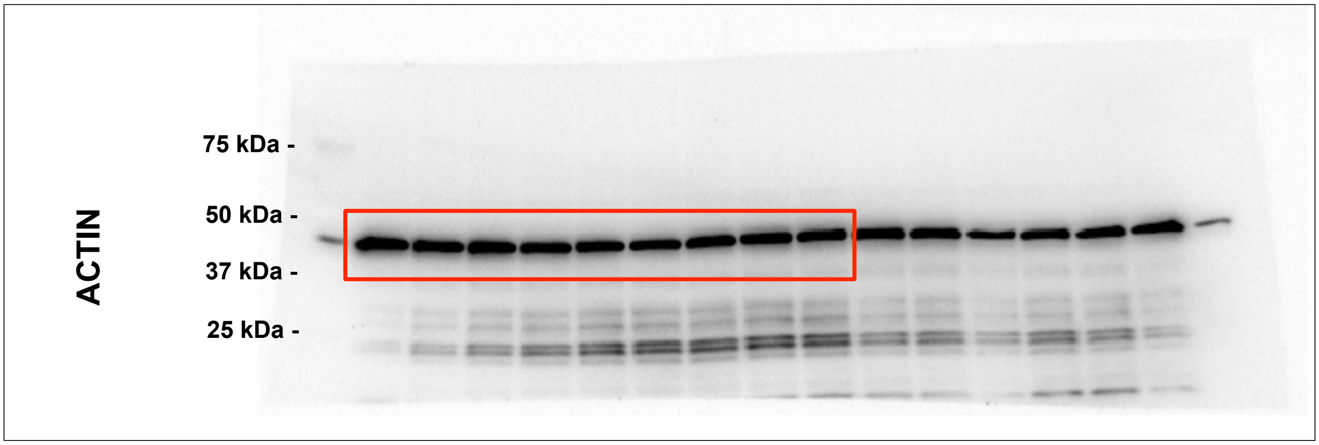

Uncropped Immunoblots for Supplementary Fig.6a

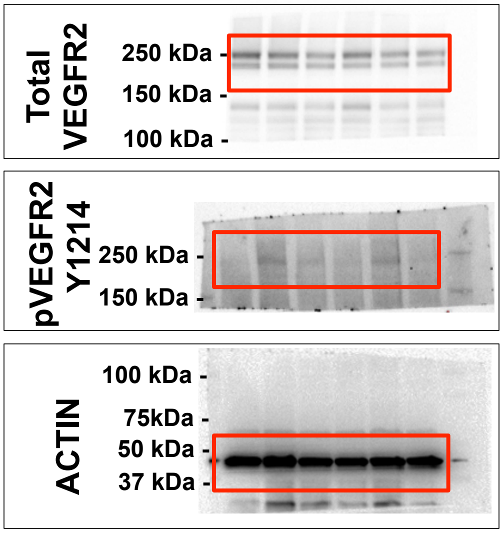

Uncropped Immunoblots for Supplementary Fig.6b

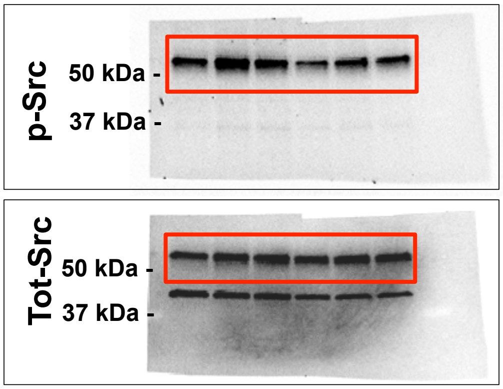

Uncropped Immunoblots for Supplementary Fig.6c

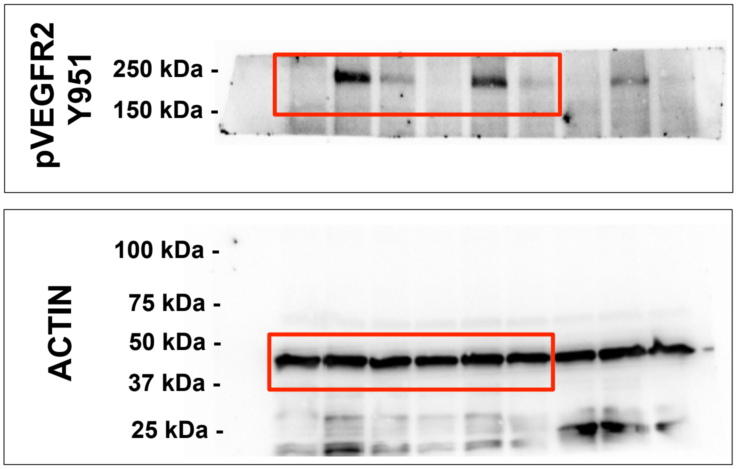

Uncropped Immunoblotsfor Supplementary Fig.7c

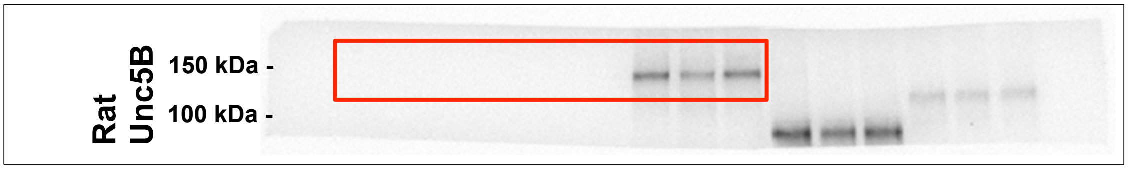

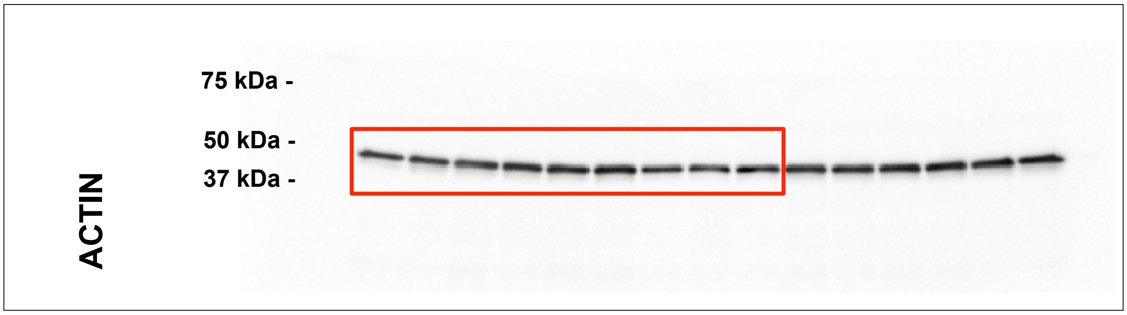

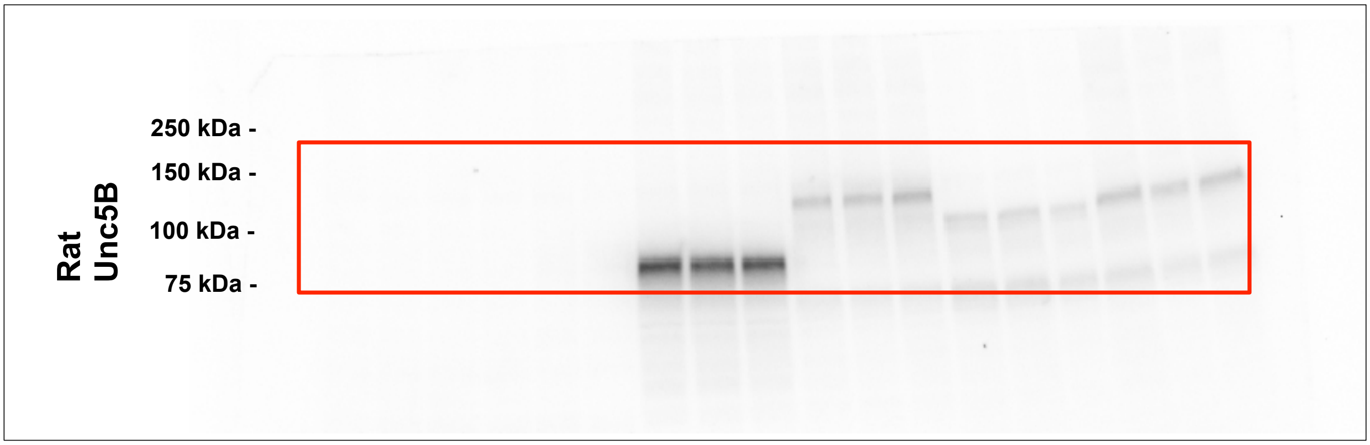

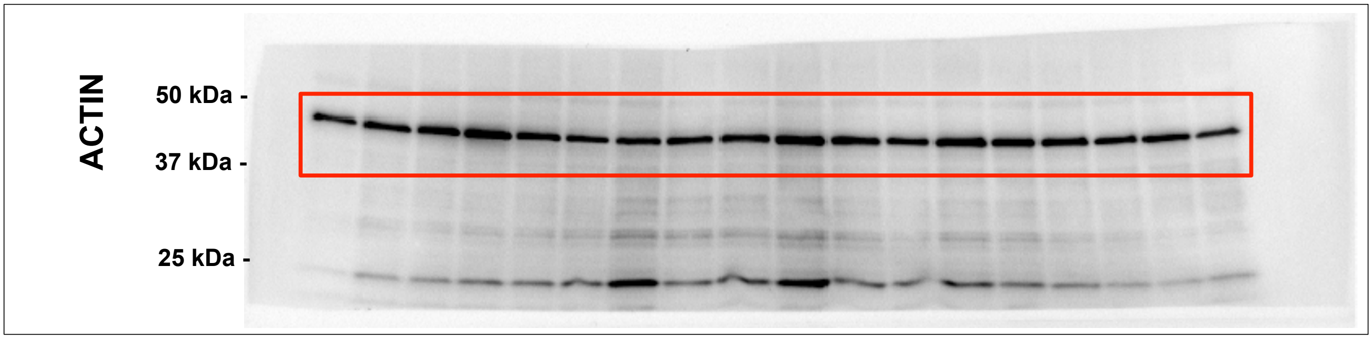

Supplementary Table 1. SiRNA targeting sequences and corresponding sequences in siRNA-resistant constructs.

siRNA Targeting Sequences	Location	siRNA-resistant Constructs			
**Human ROBO4 SMARTpool siRNAs**:		**Mouse Robo4FL**	**Mouse Robo4ΔCD**		
5'-CCTCAGAGTTCACGGACAT-3'	ECD	<70% conserved	<70% conserved		
5'-GGGCCAAGACTACGAGTTC-3'	ECD	5'-GGGCCAAGACTATGAATTC-3'	GGGCCAAGACTATGAATTC		
5'-GGGAGGATCAAGACAGCGT-3'	ICD	5'-AGGAGGATCAGGACAGCGT-3'	N.A.		
5'-TAGCTTTGGTTTCGGTCTA-3'	ICD	5'-TAGCTTTGGCCTCAGTCTG-3'	N.A.		
**Human UNC5B SMARTpool siRNAs**:		**Rat Unc5BFL**	**Rat Unc5BΔDD**	**Rat Unc5BΔUPA**	**Rat Unc5BUPA**
5'-GACCTTATGCCTTCAAGAT-3'	ICD (DD)	5'-GACCCTATGCCTTCAAGAT-3'	N.A.	N.A.	N.A.
5'-GCCCGTGACTGGATCTTTC-3'	ICD (ZU5)	5'-GCCGGAGACTGGATCTTCC-3'	5'-GCCGGAGACTGGATCTTCC-3'	5'-GCCGGAGACTGGATCTTCC-3'	N.A.
5'-GCGGATACTTGGTGGAGGA-3'	ICD (UPA)	5'-GTGGCTACTTGGTGGAGGA-3'	5'-GTGGCTACTTGGTGGAGGA-3'	N.A.	5'-GTGGCTACTTGGTGGAGGA-3'
5'-GCACATACCCTAGCGATTT-3'	ICD	5'-GTACATACCCAGGCGATTT-3'	5'-GTACATACCCAGGCGATTT-3'	5'-GTACATACCCAGGCGATTT-3'	5'-GTACATACCCAGGCGATTT-3'
**Human ROBO1 SMARTpool siRNAs:**		**Rat Robo1FL**	**Rat Robo1ΔCD**		
5'-GAATCAGACTGGTTAGTTT-3"	ECD	5'-GAATCGGAGTGGTTAGTTT-3'	5'-GAATCGGAGTGGTTAGTTT-3'		
5'-GCAGGTACTTGGAGGATAT-3'	ICD	5'-GCAAGTACTTGGGGGATTT-3'	N.A		
5'-GAGGGCAGCTAATGCATAT-3'	ECD	5'-GAGGGCAGCAAATGCCTAT-3'	5'-GAGGGCAGCAAATGCCTAT-3'		
5'-GGATGTATTTGCAACAAGA-3'	ICD	5'-GGATGTATTTGCAACAGGA-3'	N.A.		
**Human ROBO2 SMARTpool siRNAs:**					
5'-TCTCAAAGACCTCGACCTA-3'					
5'-AGTGGATGGTACAGCGTTA-3'					
5'-GCGCAATGCCAGCGACCTT-3'					
5'-GGTATGACATCAAAGACGA-3'					

Red font: Mis-matched nucleotides in siRNA-resistant constructs compared to the corresponding siRNA targeting sequences. ECD: extracellular domain; ICD: intracellular domain.

Supplementary Table 2. Genotyping and qPCR primers used in the study

Primers	Sequence	Application
GFP	Forward: 5’- CCTACGGCGTGCAGTGCTTCAGC-3’<br>Reverse: 5’-CGGCGAGCTGCACGCTGCGTCCTC-3’	Genotyping
CDH5-tTA	Forward: 5’-GACGCCTTAGCCATTGAGAT-3’<br>Reverse: 5’-CAGTAGTAGGTGTTTCCCTTTCTT-3’	Genotyping
Robo4$^{-/-}$	WT allele forward: 5’-GTCTGGATCCAAAGCCAGCAGGAC-3’<br>WT allele reverse: 5’-GGGGAGAAGGTCTGAGTCCATAGG-3’<br>KO allele forward: 5’-CAGCGCATCGCCTTCTATCGCCTT-3’<br>KO allele reverse: 5’-CACCTGTCTGTTCTCCACATCGGC-3’	Genotyping
GAPDH	Hs_GAPDH_1_SG QuantiTect Primer Assay (Qiagen, #QT00079247)	qPCR
ROBO1	Hs_ROBO1_2_SG QuantiTect Primer Assay (Qiagen, #QT01668982)	qPCR
ROBO2	Hs_ROBO2_1_SG QuantiTect Primer Assay (Qiagen, #QT00200704)	qPCR
ROBO4	Hs_ROBO4_1_SG QuantiTect Primer Assay (Qiagen, #QT00237741)	qPCR
UNC5B	Hs_UNC5B_1_SG QuantiTect Primer Assay (Qiagen, #QT00086366)	qPCR
mRobo4 (mouse Robo4ΔCD)	Forward: 5’-AATGGTGTCATCCGTGGTTAC-3’<br>Reverse: 5’-AGTTGGCAGCAGGCAATG-3’	qPCR

Supplementary Table 3. Antibodies used in the study

Antibody	Manufacturer	Cat. No.	Working Conc/Dilution	Application
β-Catenin	BD	610153	1µg/ml	Immunostaining
CD31	BD	553370	1µg/ml	Immunostaining & MLEC purification
ICAM-2	BD	553326	1µg/ml	MLEC purification
AKT	Cell Signalling	4060	1:2000	Western blot
phospho-AKT (pS473)	Cell Signalling	4691	1:2000	Western blot
Src	Cell Signalling	2109	1:2000	Western blot
phospho-Src (pY416)	Cell Signalling	6943	1:2000	Western blot
VEGFR2	Cell Signalling	9698	1:2000	Western blot
phospho-VEGFR2 (pY951)	Cell Signalling	2471	1:500	Western blot
phospho-VEGFR2 (pY1175)	Cell Signalling	2478	1:1000	Western blot
phospho-VEGFR2 (pY1214)	Cell Signalling	2477	1:300	Western blot
Unc5B	R&D	AF1065	1 µg/ml	Western blot
Robo4 (goat)	R&D	BAF2336	0.5 µg/ml<br>1µg/ml	ELISA<br>Western blot
Robo4 (human) (clone: 71.22)	Genentech	n.a	1 µg/ml	ELISA & Immunoprecipitation
GFP	Life Technologies	G10362	1:500	Immunostaining & Western blot
VE-cadherin	Santa Cruz	sc-6458	1:1000	Western blot
β-Actin	Sigma-Aldrich	A1978	1:2500	Western blot
